# Cpn60.1 (GroEL1) Contributes to Mycobacterial Crabtree Effect: Implications for Biofilm Formation

**DOI:** 10.3389/fmicb.2019.01149

**Published:** 2019-06-11

**Authors:** Sheng Zeng, Patricia Constant, Dong Yang, Alain Baulard, Philippe Lefèvre, Mamadou Daffé, Ruddy Wattiez, Véronique Fontaine

**Affiliations:** ^1^Microbiology, Bioorganic and Macromolecular Chemistry Research Unit, Faculté de Pharmacie, Université Libre de Bruxelles (ULB), Brussels, Belgium; ^2^Department of Tuberculosis and Infection Biology, Institut de Pharmacologie et de Biologie Structurale, Université de Toulouse, CNRS, UPS, Toulouse, France; ^3^Institut Pasteur de Lille, Université de Lille, CNRS, INSERM, CHU Lille, U1019-UMR 8204, Center for Infection and Immunity of Lille, Lille, France; ^4^Department of Proteomics and Microbiology, University of Mons, Mons, Belgium

**Keywords:** GroEL1, biofilm, Crabtree effect, mycobacteria, methylglyoxal, metabolic adaptation

## Abstract

Biofilm formation is a survival strategy for microorganisms facing a hostile environment. Under biofilm, bacteria are better protected against antibacterial drugs and the immune response, increasing treatment difficulty, as persistent populations recalcitrant to chemotherapy are promoted. Deciphering mechanisms leading to biofilms could, thus, be beneficial to obtain new antibacterial drug candidates. Here, we show that mycobacterial biofilm formation is linked to excess glycerol adaptation and the concomitant establishment of the Crabtree effect. This effect is characterized by respiratory reprogramming, ATP downregulation, and secretion of various metabolites including pyruvate, acetate, succinate, and glutamate. Interestingly, the Crabtree effect was abnormal in a mycobacterial strain deficient for Cpn60.1 (GroEL1). Indeed, this mutant strain had a compromised ability to downregulate ATP and secreted more pyruvate, acetate, succinate, and glutamate in the culture medium. Importantly, the mutant strain had higher intracellular pyruvate and produced more toxic methylglyoxal, suggesting a glycolytic stress leading to growth stasis and consequently biofilm failure. This study demonstrates, for the first time, the link between mycobacterial biofilm formation and the Crabtree effect.

## Introduction

Bacterial biofilms are multicellular communities comprising inhabitant cells and surrounding extracellular matrix (Hassanov et al., 2018). The biofilm-inhabiting bacteria are believed to be heterogeneous with regard to their physiology and metabolism, contributing to the survival of the biofilm community under challenging external stresses such as antibiotics (Stewart and Franklin, 2008; Moormeier et al., 2013; Liu et al., 2015; Hassanov et al., 2018). Bacterial biofilms were shown to be regulated by various stresses, such as decreased oxygen tension in *Staphylococcus aureus* (Moormeier et al., 2013) and nutritional variation in *Pseudomonas putida* (Diaz-Salazar et al., 2017). In the presence of stresses, cells within a biofilm tend to reprogram their gene transcription and protein expression in order to shape the metabolism to promote their survival or death and biofilm formation (Stewart and Franklin, 2008; Moormeier et al., 2013). As a result of metabolic adaptations, bacteria may produce some metabolites that can subsequently function as biofilm-regulating metabolic signals. This is illustrated, for example, in *Pseudomonas aeruginosa* biofilms requiring pyruvate fermentation to produce lactate (Petrova et al., 2012) and by the fact that acetic acid was shown to act as a biofilm-stimulating volatile metabolite in *Bacillus subtilis* (Chen et al., 2015). In the same organism, the glutamate/glutamine metabolism was found to contribute to biofilm formation (Liu et al., 2015; Hassanov et al., 2018). Therefore, bacterial biofilm development seems to be closely associated with metabolic adaptations. A better understanding of these metabolic shifts could help to identify vulnerable targets.

Although there is still lack of evidence for *in vivo* relevance of *Mycobacterium tuberculosis* (*M. tuberculosis*) biofilm, human lung biofilm formation by *M. abscessus* and successful *in vitro* growth of *M. tuberculosis* biofilms have been reported (Ojha et al., 2005, 2008; Qvist et al., 2015; Fennelly et al., 2016). Studies have shown that a multitude of stresses, particularly those faced by the bacilli during infection, i.e., limited oxygen availability (Ojha et al., 2008; Totani et al., 2017), accumulation of carbon dioxide (Ojha et al., 2008), and intracellular thiol reductive stress (Trivedi et al., 2016), are potent mycobacterial biofilm inducers. Despite all these, other inducing factors as well as key metabolic adaptations essential for mycobacterial biofilm formation remain largely unknown.

Nucleoid-associated proteins can regulate the formation of mycobacterial biofilms. For instance, disruption of Lsr2 leads to biofilm defect (Chen et al., 2006; Gordon et al., 2010). In addition, mycobacterial chaperonin 60.1 (Cpn60.1; also known as GroEL1), a possible nucleoid-associated protein, was also reported to be required for biofilm formation (Ojha et al., 2005, 2008; Basu et al., 2009). The role of Cpn60.1 in mycobacterial biofilm formation was mainly established in the fast-growing, saprophytic *M. smegmatis* by Ojha and colleagues, who showed that a Cpn60.1-deficient strain produced lower amounts of mycolic acids during biofilm maturation (Ojha et al., 2005). Later, they developed a *M. tuberculosis* biofilm model using standard Sauton’s medium containing 6% glycerol (Ojha et al., 2008). The role of Cpn60.1 in the regulation of biofilms of slow-growing mycobacteria may differ from that in *M. smegmatis* since Cpn60.1 is required for synthesis of phthiocerol dimycocerosates (PDIM) and phenolic glycolipids (PGL) (Wang et al., 2011; Soetaert et al., 2015), two structurally related cell wall lipids produced only in pathogenic mycobacteria and in *M. bovis* BCG (Wang et al., 2011; Yu et al., 2012; Tran et al., 2016). Hence, this study was initiated by assessing the effect of Cpn60.1 loss on biofilm formation of the slow-growing *M. bovis* BCG, a nonpathogenic surrogate for *M. tuberculosis*. After observing that the *M. bovis* BCG strain deficient for Cpn60.1 (Δ*cpn60.1*) was largely impaired for biofilm growth under standard Sauton’s medium, we intended to understand the possible mechanisms underlying this defect.

## Materials and Methods

### *M. bovis* BCG Strains and Culture Conditions

Wild-type (WT) *M. bovis* BCG, Δ*cpn60.1 M. bovis* BCG (Δ*cpn60.1*) (Wang et al., 2011), complemented *M. bovis* BCG (ComplΔ*cpn60.1*) (Wang et al., 2011), WT *M. bovis* BCG (Pasteur 1173p2 strain), PDIM^−^/PGL^+^ PMM137 strain (Simeone et al., 2010), and PDIM^−^/PGL^−^ PMM50 strain (Astarie-Dequeker et al., 2009) were described previously. In particular, the Δ*cpn60.1* strain was constructed by allelic gene exchange to insert a kanamycin resistance-conferring cassette into the *cpn60.1* gene. The ComplΔ*cpn60.1* strain was constructed by introduction into the Δ*cpn60.1* strain of a mycobacteriophage MS6-derived integrative vector that expresses Cpn60.1 under the control of its own promoter and a hygromycin resistance marker (Wang et al., 2011). The Δ*cpn60.1* and PMM50 strains were grown in the presence of 25 μg/ml kanamycin. The ComplΔ*cpn60.1* strain was grown in media with 25 μg/ml kanamycin and 50 μg/ml hygromycin B.

### Biofilm Growth

Mycobacterial precultures in Dubos Tween Albumin (DTA) medium at an OD600 of 0.9 were diluted 100-fold in Sauton’s medium in six-well plates or petri dishes to grow biofilms. The plates or petri dishes, tightly closed using parafilm in order to allow progressive accumulation of carbon dioxide and the decrease of oxygen concentration (Ojha et al., 2008), were incubated for up to 35 days at 37°C. At indicated time points, biofilm cultures were opened for visual check and for photographing with a digital camera (Sony). One liter of standard Sauton’s medium contains 0.5 g of KH_2_PO_4_, 0.5 g of MgSO_4_, 4 g of L-asparagine, 2 g of citric acid, 0.05 g of ferric ammonium citrate, 60 ml of glycerol (6%, v/v), and 0.0001% (w/v) ZnSO_4_ (Kulka et al., 2012). For mixed WT and Δ*cpn60.1* biofilm growth, 100 μl of each strain (washed to remove kanamycin for the mutant preculture) was inoculated in 20 ml of standard biofilm medium.

### Normal Growth Assay

BCG precultures in DTA medium (OD600 of 0.8–1) were pelleted and washed in 0% glycerol Sauton’s medium and subcultured in Sauton’s medium containing various glycerol concentrations. In some experiments, 1:1 mixture of the Δ*cpn60.1* and WT strains was inoculated. After 9 days of growth, pictures were taken. To quantify growth, clumped cells were disrupted in 7H9/0.05% Tween 80 with 3 mm glass beads before OD600 and viability measurement.

### Methylglyoxal Susceptibility Assay

Minimal inhibitory concentrations (MICs) inhibiting 99% mycobacterial growth were determined in Middlebrook 7H9/0.2% glycerol/10% albumin–dextrose–catalase (ADC) enrichment. Briefly, exponential precultures (OD600 of 0.3–0.5) were inoculated into 1 ml of medium with twofold serial methylglyoxal (MG) dilutions ranging from 20 to 0.078 mM, yielding a starting inoculum of 5 × 10^5^ CFU/ml. The results were recorded when growth of a MG-free 1% inoculum control became visible (normally after 5–6 days of growth depending on experiments).

### Determination of Methylglyoxal–Protein Adduct by Enzyme-Linked Immunosorbent Assay

Cells were pelleted and resuspended in 300 μl of 100 mM bicarbonate/carbonate coating buffer (pH 9.6), followed by sonication in Bioruptor UCD-200. The lysates were cleared by centrifugation, and the supernatant was coated for enzyme-linked immunosorbent assay (ELISA). The reagents for this assay, including primary antibody targeting MG hydroimidazolone protein adduct (MG-H1) and secondary antibody horseradish peroxidase (HRP) conjugate, were all included in an OxiSelect MG competitive ELISA kit (Cell Biolabs). The ELISA was performed according to the manual. Absorbance at 450 nm was recorded using a plate reader and normalized as per protein concentrations determined by the Bradford assay.

### Determination of ATP

Bacterial ATP was determined using a BacTiter-Glo Microbial cell viability assay kit (Promega). Briefly, 20 μl of bacterial culture was mixed with equal volume of the BacTiter-Glo reagent for 5 min in the dark. Luminescence was subsequently recorded using Lumat LB 9507 (Berthold).

### Quantification of Pyruvate, Succinate, Acetate, and Glutamate/Glutamine

Pyruvate assay kit (Abnova), succinate colorimetric assay kit (BioVision), and acetate colorimetric assay kit (Sigma) were used for metabolite quantification. Procedures were followed according to the manuals. *M. bovis* BCG was grown in 6% glycerol Sauton’s medium for 9 or 11 days before culture filtrates were harvested for extracellular metabolite quantification. In some experiments, cells were pelleted and lysed by sonication prior to intracellular metabolite quantification. The concentrations of these metabolites were calculated based on their corresponding standard curves and normalized by protein concentrations.

Glutamate/glutamine was measured using the glutamine/ glutamate-Glo^TM^ assay kit (Promega) according to the manual. The glutamate/glutamine level [measured as relative light unit (RLU)] was recorded by Lumat LB 9507. Data were normalized by OD600.

### Proteomic Analysis

WT and Δ*cpn60.1* BCG strains were grown as biofilms for 25 days in 4% glycerol Sauton’s medium for proteomic comparison. Alternatively, WT BCG was grown as biofilm cultures for 25 days in 0.2, 2, 4, 6, and 8% glycerol Sauton’s medium for proteomic analysis. Protein extraction was described previously (Deschoenmaeker et al., 2017). Detailed proteomic procedure can be found either in the Supplementary Material or in our previous publication (Zeng et al., 2019). Protein hits with a *p*< 0.05 and a fold change <0.8 or >1.2 were further analyzed. Protein function predication was based on Mycobrowser^1^, NCBI Conserved Domains search tool^2^, and UniProt^3^. Among the analyzed proteins, proteins with unknown function are not included in Supplementary Tables S1, S2. However, these proteins can be found in the supplementary Excel file named “Original proteomic data”.

### Lipid Analysis

Twenty five days BCG biofilm cultures in 4% glycerol Sauton’s medium were subjected to lipid extraction as described previously (Camacho et al., 2001; Constant et al., 2002; Simeone et al., 2010). An equivalent amount of the extracted lipids was spotted onto thin-layer chromatography (TLC) plates and separated with petroleum ether/diethyl ether (90/10, v/v) for PDIM analysis and with CHCl_3_/HC_3_OH (95/5, v/v) for PGL analysis. Lipids were visualized by spraying the plates with 10% phosphomolybdic acid in ethanol for PDIM, and 0.2% anthrone in concentrated H_2_SO_4_ (w/v) for PGL.

### Statistical Analysis

Figures were prepared using GraphPad prism 6.0. Unless otherwise specified, unpaired *t*-test performed in GraphPad prism 6.0 was applied for statistical analysis. In the case of significantly different variances, unpaired *t*-test with Welch’s correction was used. A *p*< 0.05 was considered as statistically significant. Depending on graphs, pooled data from at least three independent experiments or data from one representative experiment (done in triplicate) are shown.

## Results

### Defective PDIM and PGL Production Partially Contributes to Biofilm Defect of the Δ*cpn60.1 M. bovis* BCG

**FIGURE 1:**
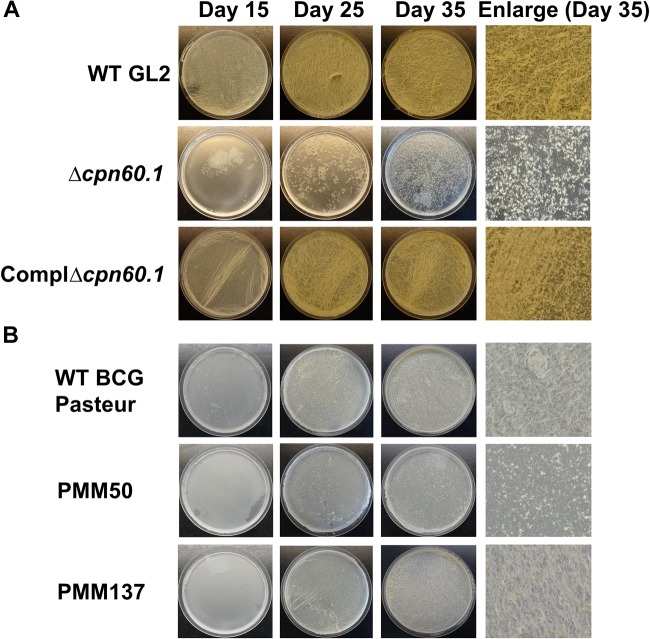
The Δ*cpn60.1* biofilm defect under standard Sauton’s medium partially resulted from PDIM/PGL alteration. **(A)** BCG GL2 strains were grown as biofilms in 6% glycerol Sauton’s medium for indicated time points. The ComplΔ*cpn60.1* is the complemented strain expressing Cpn60.1. **(B)** Biofilms of BCG Pasteur strains were grown as in **(A)**. PMM50 and PMM137 are PDIM^−^/PGL^−^ and PDIM^−^/PGL^+^, respectively. The experiments were performed at least three times. Representative biofilm pictures are shown.

The role of Cpn60.1 on mycobacterial biofilm growth was mainly established in *M. smegmatis* (Ojha et al., 2005). Here, we assessed it with the Cpn60.1-deficient *M. bovis* BCG mutant. This mutant, where the *cpn60.1* gene is disrupted by a kanamycin resistance-conferring cassette, was previously generated by the allelic gene exchange method (Wang et al., 2011). In the standard Sauton’s medium containing 6% glycerol, the Δ*cpn60.1 M. bovis* BCG strain (Δ*cpn60.1*) displayed very poor biofilm phenotype with no full attachment on the medium–air interface at day 35 (Figure 1A). On the opposite, fully matured wild-type (WT) biofilms appeared showing ridges and troughs (Figure 1A; Ojha et al., 2005). Importantly, the biofilm defect was restored in the complemented strain expressing Cpn60.1 (Figure 1A), corroborating that the loss of Cpn60.1 was responsible for the biofilm defect.

In 6% glycerol Sauton’s medium, the Δ*cpn60.*1 strain produces no PDIM and a markedly reduced amount of PGL in the cell wall (Wang et al., 2011; Soetaert et al., 2015). To test whether PDIM/PGL alteration could account for the Δ*cpn60.1* strain biofilm defect under this condition, we examined the biofilm phenotype of the PDIM^−^/PGL^−^ PMM50 (Δ*ppsE*) strain (Astarie-Dequeker et al., 2009) and of the PDIM^−^/PGL^+^ PMM137 (Δ*fadD26*) strain (Simeone et al., 2010). As shown in Figure 1B, although loss of only PDIM had a negligible effect on mycobacterial biofilm formation, absence of both lipids reduced the biofilm maturation (Figure 1B). These results suggested a role for PDIM/PGL in mycobacterial biofilm maturation. Considering that the biofilm defect of the Δ*cpn60.1* strain was stronger than that of the PDIM^−^/PGL^−^ strain, the Δ*cpn60.1* biofilm failure could be only partially linked to its PDIM/PGL alteration.

### Poor Growth Contributes Largely to the Δ*cpn60.1* Biofilm Defect in Excess Glycerol Culture Condition

**FIGURE 2:**
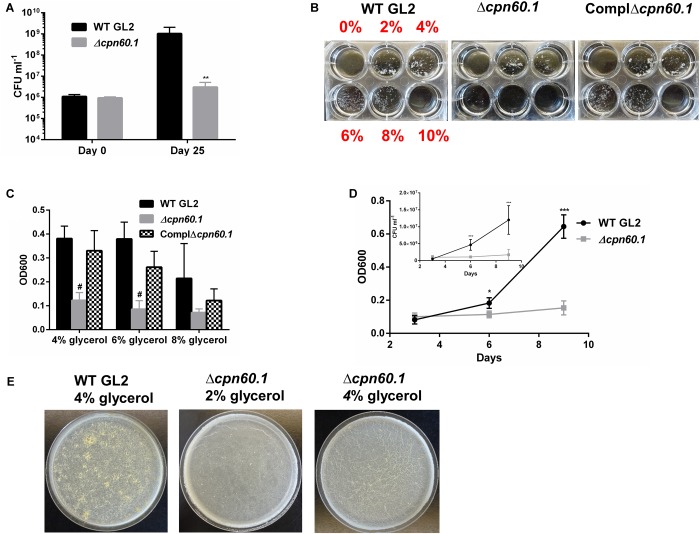
The Δ*cpn60.1* BCG strain was more susceptible to excess glycerol present in the biofilm medium. **(A)** Viability of biofilm culture under 6% glycerol Sauton’s medium at days 0 and 25 was determined. ^∗∗^*p* ≤ 0.01 relative to WT at day 25 by unpaired *t*-test. **(B)** Washed BCG precultures were subcultured in Sauton’s medium with various glycerol concentrations as indicated for 9 days. Representative pictures are shown. **(C)** Cells grown in 4, 6, and 8% glycerol Sauton’s medium were disrupted before OD600 determination. ^#^*p* ≤ 0.0001 relative to WT by unpaired *t*-test. **(D)** Growth kinetics under 6% glycerol Sauton’s medium measured by OD600 and viability (inset) at 3, 6, and 9 days. ^∗^*p* ≤ 0.05; ^∗∗∗^*p* ≤ 0.001 by unpaired *t*-test. **(E)** Representative 25 days biofilms grown in Sauton’s medium with 2 and 4% glycerol as indicated.

In our attempts to grow biofilm with the Δ*cpn60.1* strain, we repeatedly noticed that the growth of this strain under the standard biofilm condition seemed very poor (Figure 1A). We therefore compared the biofilm culture viability of the WT and Δ*cpn60.1* strains at 25 days by CFU counting. The defective Δ*cpn60.1* biofilm culture contained approximately 3 log less bacilli than the WT BCG biofilm (Figure 2A). Relative to the inoculation size, the biofilm culture viability of the mutant strain only showed a slight increase in CFU counting (i.e., 9.37 × 10^5^ at day 0 versus 3.02 × 10^6^ at day 25), indicating poor growth. This severely compromised growth of the Δ*cpn60.1* strain under the standard biofilm medium could therefore be the main cause of the Δ*cpn60.1* biofilm growth defect.

Given that the relatively high glycerol concentration (i.e., 6%) in the standard Sauton’s medium is likely to generate a glycolytic stress under which the bacilli could necessitate Cpn60.1 to maintain its fitness, we compared the growth rate of the WT, the Δ*cpn60.1*, and the complemented strains in Sauton’s medium with various glycerol concentrations. Consistent with the biofilm growth defect (Figure 1A, 2A), the growth of the Δ*cpn60.1* strain was extremely poor under 6% glycerol Sauton’s medium, a phenotype restored in the complemented strain (Figure 2B,C). The growth kinetic under 6% glycerol Sauton’s medium measured by OD600 and CFU counting revealed that although comparable to WT at day 3, the Δ*cpn60.1* strain encountered growth stasis thereafter (Figure 2D). Interestingly, we observed improved growth of the Δ*cpn60.1* strain under lower glycerol concentrations (Figure 2B), indicating that this strain is able to catabolize glycerol. It is worth noting that the growth of PMM50 and PMM137 was comparable to their parental WT strain regardless of the glycerol concentration (Supplementary Figure S1 and data not shown). Thus, loss of Cpn60.1 renders the bacilli more susceptible to the excess glycerol independently of PDIM/PGL alteration.

We next assessed if the Δ*cpn60.1* strain was able to grow biofilm under growth-permissive conditions by reducing the glycerol concentration. Importantly, the Δ*cpn60.1* biofilm was improved in 2% and, markedly, in 4% glycerol Sauton’s medium (Figure 2E), in agreement with the improved growth under these conditions (Figure 2B). Since slow-growing mycobacteria are believed to “uptake” glycerol passively (Pacifico et al., 2018), a larger extracellular glycerol concentration (e.g., 6%) may result in higher intracellular concentration of this molecule. This could conceivably generate a stronger glycolytic stress that exceeds the ability of the mutant strain to adapt. Under 4% glycerol Sauton’s medium, the Δ*cpn60.1* biofilm showed a less matured phenotype than that of WT BCG (Figure 2E), likely due to the decreased amount of PDIM/PGL in the cell wall (Supplementary Figure S2). Taken together, these results strongly demonstrate that poor growth of the Δ*cpn60.1* strain accounted largely for its biofilm failure under 6% glycerol Sauton’s medium.

### Accumulation of Methylglyoxal Accounts for the Δ*cpn60.1* Growth Defect

**FIGURE 3:**
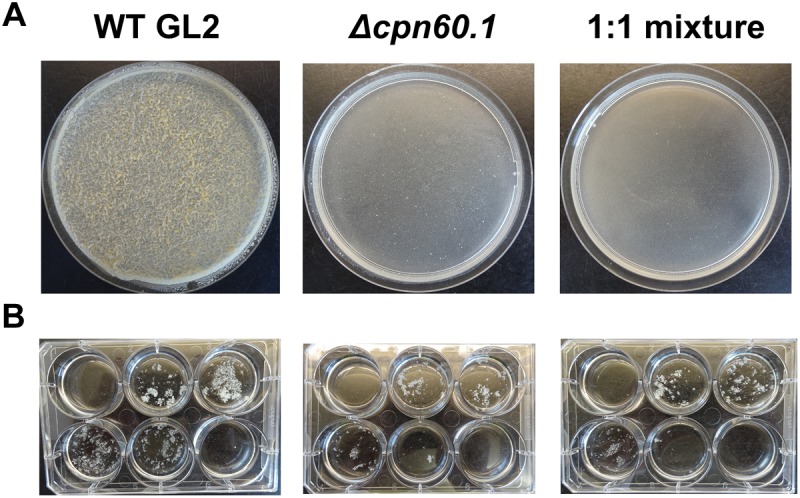
The Δ*cpn60.1* strain inhibited WT biofilm and normal growth. **(A)** Representative 25 days biofilms under 6% glycerol Sauton’s medium are shown. **(B)** Growth of BCG strains in Sauton’s medium with varying glycerol concentrations as in Figure 2B. The experiments were performed at least three times.

Since the growth inhibition associated with glycerol did not occur in the early phase (i.e., day 3 of growth) (Figure 2D), we anticipated that the initial glycerol uptake and catabolism were not disturbed in the mutant and that inhibition in the latter growth phase was associated with some toxic metabolite(s) produced in the glycerol catabolism pathway. Interestingly, coculture of the Δ*cpn60.1* and WT strains abrogated WT biofilm and normal growth (Figure 3A,B). This observation supported our hypothesis that the Δ*cpn60.1* strain produced some metabolite(s) inhibiting the growth of the WT strain under standard Sauton’s medium.

**FIGURE 4:**
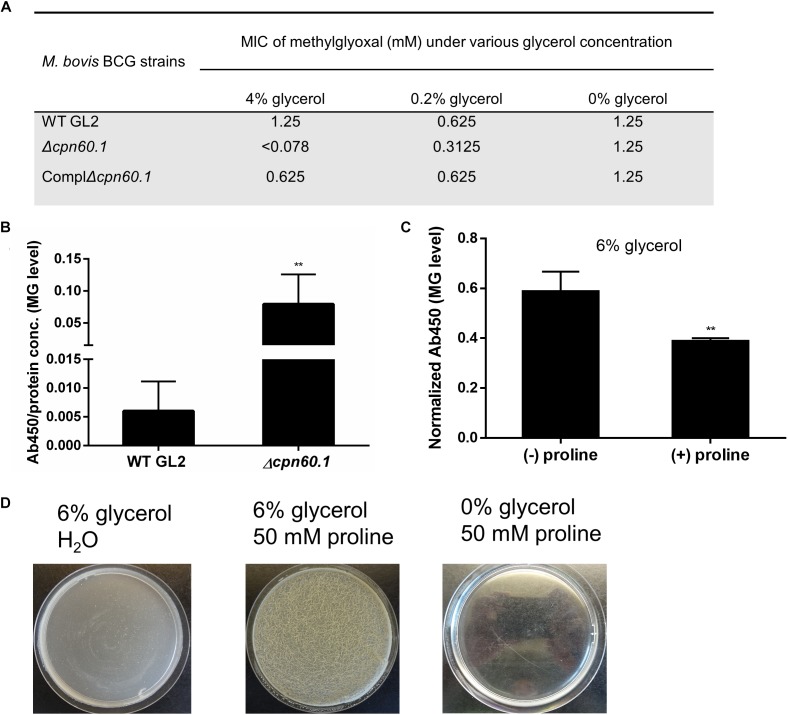
The Δ*cpn60.1*’s accumulation of methylglyoxal and rescue of its biofilm by proline-mediated detoxification. **(A)** MIC of methylglyoxal determined in 7H9 medium with varying amounts of glycerol. **(B)** Cellular extracts of BCG grown in 6% glycerol Sauton’s medium were determined for methylglyoxal–protein adducts by ELISA. Absorbance at 450 nm was normalized by dividing by protein concentration. ^∗∗^*p* ≤ 0.01 relative to WT by unpaired *t*-test. **(C)** WT BCG protein extracts from 6% glycerol Sauton’s medium (±25 mM proline) were quantified for methylglyoxal, followed by data normalization as per protein concentration. ^∗∗^*p* ≤ 0.01 by unpaired *t*-test. **(D)** The Δ*cpn60.1* biofilm was grown in 6% glycerol Sauton’s medium for 25 days with or without 50 mM proline. A control with proline and without glycerol was included.

Glycerol is first catabolized *via* glycolysis, which, through hydrolysis of triose phosphates, can give rise to MG, a toxic by-product capable of reacting with macromolecules such as proteins (Ferguson et al., 2000; Pethe et al., 2010). To investigate if the growth defect of the Δ*cpn60.1* strain (Figure 2) was associated with MG accumulation or higher susceptibility to MG, we first determined the MIC of MG. Without glycerol, all tested strains exhibited identical MG susceptibility regardless of the presence of Cpn60.1. However, the Δ*cpn60.1* strain exclusively became 2- and 16-fold more susceptible to the added MG in 0.2 and 4% glycerol medium, respectively (Figure 4A), indicating that loss of Cpn60.1 leads to more endogenous MG generation and/or compromised MG defense under excess glycerol.

To assess if MG accumulated in the absence of Cpn60.1, we quantified by ELISA the amount of MG hydroimidazolone protein adduct (MG-H1), a representative form of MG–protein mo-difications (Ahmed et al., 2003). As expected, the Δ*cpn60.1* strain accumulated significantly more MG-H1 than the WT strain under 6% glycerol Sauton’s medium (Figure 4B). To further verify that the Δ*cpn60.1* growth and biofilm defect indeed resulted from MG accumulation, we tested whether the addition of proline, previously proposed to mediate MG detoxification (Berney et al., 2012), could rescue the Δ*cpn60.1* biofilm growth. Importantly, the addition of proline, able to reduce the amount of MG-H1 (Figure 4C), markedly improved the Δ*cpn60.1* growth and biofilm development under 6% glycerol Sauton’s medium (Figure 4D and data not shown), demonstrating that the growth and biofilm defect of the Δ*cpn60.1* strain under standard Sauton’s medium mainly resulted from MG-associated stress.

### Problematic Crabtree Effect in the Δ*cpn60.1* Strain

**FIGURE 5:**
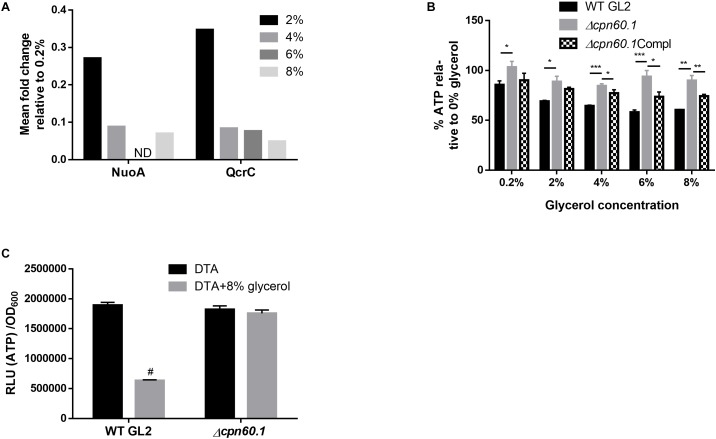
Requirement for Cpn60.1 in the glycerol-triggered ATP downregulation. **(A)** Mean fold decrease of mycobacterial respiratory proteins NuoA and QcrC relative to 0.2% glycerol Sauton’s medium. ND, no data due to a *p*> 0.05. **(B)** BCG strains grown in DTA medium were treated with glycerol for 24 h before ATP measurement. Percentage of ATP decrease was calculated by comparing with 0% glycerol control. ^∗^*p* ≤ 0.05; ^∗∗^*p* ≤ 0.01 and ^∗∗∗^*p* ≤ 0.001 by unpaired *t*-test. **(C)** BCG strains were grown in DTA medium ( ± 8% glycerol) to exponential phase before ATP measurement. ^#^*p* ≤ 0.0001 relative to WT (no glycerol) by unpaired *t*-test.

Two main pathways, i.e., glycolysis and oxidative phosphorylation, generate ATP, with oxidative phosphorylation contributing predominantly. However, the two pathways are never side by side fully active in a single cell (Sokolov et al., 2015). It has been reported, in several organisms, that under high glycolytic substrates, glycolysis is enhanced, favoring biomass synthesis, while aerobic oxidative phosphorylation is suppressed, a phenomenon known as the Crabtree effect (Crabtree, 1929; Kosmachevskaya et al., 2015). To our knowledge, such an effect has not been reported in mycobacteria. If mycobacteria display the aforementioned Crabtree effect, the oxidative phosphorylation should be downregulated under excess glycerol. Interestingly, proteomic analysis revealed that relative to 0.2% glycerol Sauton’s medium, WT BCG cultured with higher glycerol concentrations downregulated NuoA, a component of the proton-pumping type I NADH dehydrogenase (NDH-I), and QcrC, a constituent of the energy-efficient cytochrome *bc_1_*/*aa_3_* branch of the electron transport chain (ETC) (Shi et al., 2005; Lu et al., 2015; Figure 5A). This suggested ETC downregulation and a possible lower energy state under excess glycerol. Indeed, the WT BCG quickly decreased its ATP production upon glycerol treatment. In stark contrast, the glycerol-triggered ATP downregulation was significantly compromised in the Δ*cpn60.1* strain (Figure 5B), indicating a problematic ETC reprogramming upon Cpn60.1 loss. Importantly, the mutant strain grown under excess glycerol produced significantly more ATP than the WT strain (Figure 5C). Therefore, the Crabtree effect in the mutant strain was problematic.

In addition to the respiratory downregulation, the Crabtree effect is also characterized by glycolytic enhancement (Kosmachevskaya et al., 2015). Proteomic analysis of WT BCG grown in 6 and 0.2% glycerol Sauton’s medium indeed revealed upregulation of various glycolytic enzymes, e.g., glycerol kinase (Glpk; 4.3-fold) and pyruvate kinase (PykA; 1.6-fold) (Supplementary Table S1), suggesting enhancement of glycolysis under excess glycerol. This could lead to enhanced production of pyruvate, the glycolytic end product.

**FIGURE 6:**
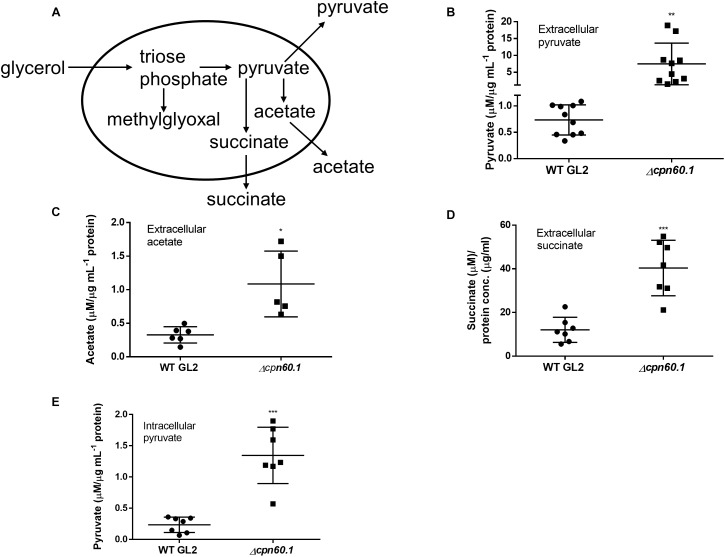
The Δ*cpn60.1* strain secreted more pyruvate, acetate, and succinate. **(A)** Proposed pathways generating methylglyoxal, pyruvate, acetate, and succinate. The secretion of pyruvate, acetate, and succinate under excess glycerol is indicated. **(B)** Pyruvate in the culture filtrates from 6% Sauton’s medium was quantified and normalized by protein concentration. ^∗∗^*p* ≤ 0.01 relative to WT by unpaired *t*-test. **(C)** Extracellular acetate was normalized by protein concentration. ^∗^*p* ≤ 0.05 by unpaired *t*-test. **(D)** Succinate in the culture filtrates from 6% Sauton’s medium was quantified and normalized by protein concentration. ^∗∗∗^*p* ≤ 0.001 by unpaired *t*-test. **(E)** Intracellular pyruvate was measured and normalized by protein concentration. ^∗∗∗^*p* ≤ 0.001 by unpaired *t*-test.

The fulfillment of Crabtree effect necessitates secretion of metabolites, such as ethanol, lactate, and acetate depending on organisms, to limit accumulation of pyruvate (Paczia et al., 2012; Sokolov et al., 2015). As nonfermentive bacteria, pyruvate may be converted to acetate in mycobacteria as demonstrated previously (Figure 6A; Rucker et al., 2015). In addition, secretion of pyruvate (Lee et al., 2013) and of succinate *via* reductive tricarboxylic acid cycle (TCA; Figure 6A; Watanabe et al., 2011) were also described for mycobacteria. We reasoned that mycobacterial Crabtree effect requires secretion of these metabolites. As expected, we observed the presence of pyruvate, acetate, and succinate in the culture filtrate under excess glycerol (Figure 6B–D). In contrast, very limited secretion of these metabolites occurred when cells were grown under DTA medium (data not shown). Importantly, the mutant strain secreted more of these metabolites (Figure 6B–D). These results further point to a problematic Crabtree effect in the absence of Cpn60.1 and indicate that the mutant strain may produce a more glycolytic end product under excess glycerol. To ascertain this, we quantified intracellular pyruvate and noticed that the Δ*cpn60.1* strain indeed accumulated significantly more pyruvate intracellularly (Figure 6E). This overaccumulation may conceivably lead to overabundance of upstream metabolites including triose phosphates, potentially explaining the MG overproduction (Figure 6A).

### Proteomic Analysis

In order to uncover events related to WT biofilm development and mycobacterial adaptation under excess glycerol, we compared proteomics of WT *M. bovis* BCG biofilms (25 days) grown under 6 and 0.2% glycerol Sauton’s medium (Supplementary Table S1). A total of 377 proteins were differentially expressed. Ten (putative) methyltransferases were upregulated. Interestingly, proteins required for biosynthesis of PDIM/PGL, e.g., Pps proteins, and of mycolic acids, e.g., KasAB and HadABC, were elevated in 6% glycerol medium, suggesting cell wall remodeling under excess glycerol and biofilm growth. Interestingly, we also observed upregulation of fatty acid-CoA ligases FadD1, 7, 10, and 11, enoyl-CoA hydratases EchA3 and 6, and acyl-CoA dehydrogenase FadE16, and downregulation of FadD9 and FadE10. Furthermore, expression of the ESX-5 type VII secretion system, required for mycobacterial fatty acid uptake (Ates et al., 2015), was significantly increased. These observations suggest that under excess glycerol and/or during biofilm growth, mycobacteria may acquire and catabolize selective fatty acids. Additionally, components of ESX-2 (EspG_2_ and MycP_2_) and the ESX-3 type VII secretion system (EccA_3_) were also upregulated. Interestingly, EspR, a previously described nucleoid-associated protein that could regulate ESX-1, ESX-2, ESX-5, and the PDIM locus (Blasco et al., 2012), was also enhanced (1.53-fold), suggesting a pivotal role for this protein during mycobacterial biofilm formation. We also observed downregulation of IdeR (0.61-fold), an iron-dependent repressor (Pandey and Rodriguez, 2014), and upregulation of proteins involved in iron storage (bacterioferritin, BfrA, 2.09-fold) and in the synthesis of iron-containing heme (i.e., Rv0260c, uroporphyrinogen-III synthase, 2.99-fold; and HemA, glutamyl-tRNA reductase, 1.46-fold), suggesting that iron and heme metabolism plays a role in mycobacterial biofilm formation. In addition to these changes, expression of thioredoxins, peroxiredoxins, thioredoxin reductases, the NADPH-generating pentose phosphate pathway, and, notably, a putative MG-detoxicating glyoxalase I (Rv0911) was significantly induced (22-fold), reflecting enhancement of mycobacterial stress tolerance and detoxification under excess glycerol.

**FIGURE 7:**
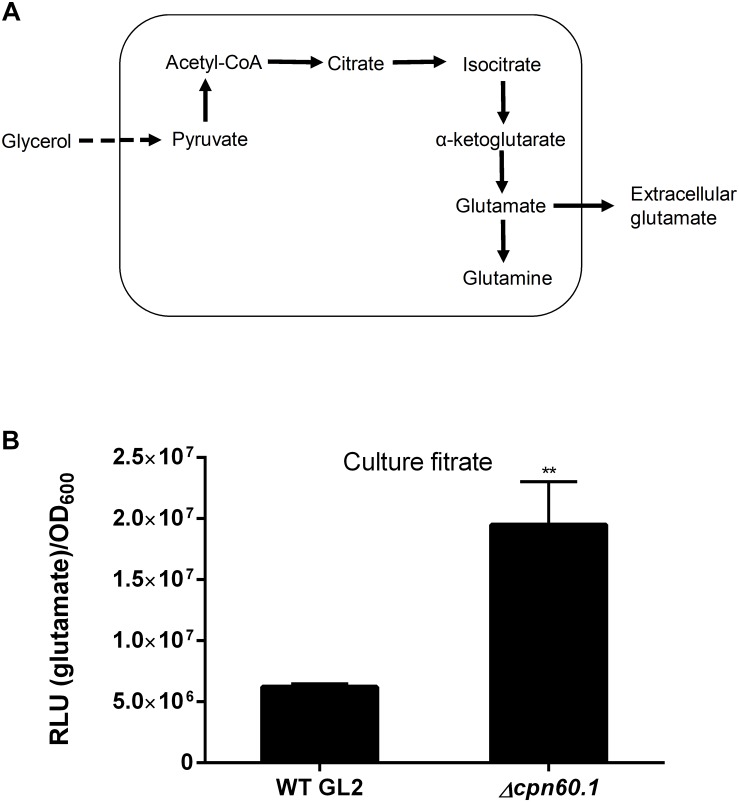
The Δ*cpn60.1* strain secreted more glutamate than the WT strain. **(A)** Proposed pathway leading to glutamate/glutamine production and the subsequent secretion of glutamate. **(B)** *M. bovis* BCG was grown in 6% glycerol Sauton’s medium for 11 days, and the culture filtrates were determined for glutamate. The experiments were performed three independent times (each in three or four biological replicates). Data from one representative experiment are shown. ^∗∗^*p* < 0.01 relative to WT by unpaired *t*-test.

As mentioned above, proteins involved in glycolysis, e.g., glycerol kinase (4.3-fold) and pyruvate kinase (1.6-fold), were upregulated under excess glycerol, suggesting an enhanced glycolytic flux. Furthermore, mycobacteria also enhanced the expression of pyruvate dehydrogenase complex (AceE; 1.4-fold), indicating that the conversion of pyruvate to acetyl-CoA is important for adaptation under excess glycerol. Notably, the phosphate acetyltransferase (Pta; 1.5-fold) catalyzing the conversion of acetyl-CoA to acetyl phosphate was upregulated, pointing to activation of the Pta-acetate kinase (AckA) pathway required for acetate production from pyruvate under excess glycerol (Figure 6A; Rucker et al., 2015). This notion is validated by secretion of acetate for both strains under excess glycerol (Figure 6C). In addition, TCA enzymes, such as fumarate hydratase (1.5-fold), malate dehydrogenase (3.2-fold), and citrate synthase (1.5-fold) were upregulated under excess glycerol. In contrast, the α-ketoglutarate decarboxylase (Kgd; 0.57-fold) was downregulated, suggesting inefficient α-ketoglutarate oxidation. Therefore, mycobacteria may employ both reductive (leading to production of succinate) (Figure 6A) and oxidative (leading to production of α-ketoglutarate) (Figure 7A) TCA branches simultaneously. The activation of reductive TCA was supported by the secretion of succinate for both strains (Figure 6D; Watanabe et al., 2011). Intriguingly, in addition to these metabolic adaptations, we also observed an augmented expression of NADH-dependent glutamate synthase (GltD; 2.7-fold), glutamine synthetase (GlnA1; 2.2-fold), and glutamine ABC transporter (GlnQ; 2.4-fold). The activation of this alternative α-ketoglutarate-assimilating pathway not only further supports the inefficiency of α-ketoglutarate oxidation but also suggests a possible glutamate/glutamine flux under excess glycerol and during biofilm formation.

To understand better the role of Cpn60.1, proteomic analysis was performed for the WT and Δ*cpn60.1* strain biofilm cultures in 4% glycerol Sauton’s medium (Supplementary Table S2). Relative to WT BCG, proteins encoded in the PDIM/PGL locus (Camacho et al., 2001) (e.g., PpsC) were significantly decreased in the absence of Cpn60.1, in good agreement with the defective PDIM/PGL production (Supplementary Figure S2). In addition, proteins involved in mycolic acid biosynthesis seemed to be differentially regulated in the mutant strain as evidenced by upregulation of Fas and FadD32 but downregulation of DesA1, possibly reflecting the previously reported alteration of mycolic acid in the defective Δ*cpn60.1 M. smegmatis* biofilm (Ojha et al., 2005). Interestingly, the expression of the DosR regulon, including the sensor kinase DosS (Park et al., 2003), was significantly decreased in the Δ*cpn60.1* strain, suggesting the requirement for Cpn60.1 in the DosR regulon induction. The DosR and its regulon may be important for mycobacterial fitness and survival under stress conditions (e.g., hypoxia) (Boon and Dick, 2002).

### The Δ*cpn60.1* Strain Secreted More Glutamate

The enhanced expression of GltD, GlnA1, and GlnQ under standard biofilm medium suggests that the glutamate/glutamine flux may be activated, contributing to the fulfillment of Crabtree effect through pyruvate assimilation as proposed in Figure 7A. We reasoned that the produced glutamate and/or glutamine could be further secreted. As expected, glutamate was found in the culture filtrates of both WT and the Δ*cpn60.1* strain grown under 6% glycerol Sauton’s medium. The mutant strain secreted significantly more of this metabolite (Figure 7B), as is the case for pyruvate, acetate, and succinate (Figure 6), thus reflecting again the higher accumulation of pyruvate in the mutant strain (Figure 6E). In contrast to glutamate, no or very limited amount of glutamine was secreted for both strains (data not shown). To the best of our knowledge, the glutamate/glutamine flux and the glutamate secretion have not been reported before as part of the Crabtree effect.

## Discussion

In the current study, we investigated for the first time the role of Cpn60.1 on biofilm formation in slow-growing mycobacteria. Under standard biofilm medium, the failure of Δ*cpn60.1* biofilm development is largely due to poor growth associated with MG overproduction. Indeed, the growth of Δ*cpn60.1* biofilm was largely restored by the addition of proline, which contributed to MG detoxification. As a highly reactive dicarbonyl compound, MG is well known to damage proteins and DNAs, leading to the formation of advanced glycation end products and, consequently, carbonyl stress (Pethe et al., 2010; Kosmachevskaya et al., 2015). While a high level of MG (e.g., added exogenously at 2 mM) led to bacillary death (data not shown), the growth stasis we observed for the Δ*cpn60.1* strain under standard biofilm medium suggests that the carbonyl stress encountered by this strain was likely less than that required for lethal effect. However, we could not rule out an equilibrium of cell division and death for the mutant strain. Several studies have demonstrated the toxic effect of MG in mycobacteria. For instance, the previously reported glycerol-dependent antimicrobial activity of pyrimidine–imidazole compounds was linked to MG cytotoxicity (Pethe et al., 2010). In addition, the activity of pretomanid, a clinical phase III antimycobacterial drug candidate, may also be linked to MG overproduction (Baptista et al., 2018).

We next aimed to investigate mechanisms underlying MG overabundance in the Δ*cpn60.1* strain. MG generation can occur as a result of triose phosphate accumulation (Figure 6A; Pethe et al., 2010). In this study, we observed that under growth conditions using standard biofilm medium, the mutant strain produced more pyruvate, the glycolytic end product. Furthermore, the mutant strain secreted significantly more of metabolites (e.g., acetate and glutamate) that could be produced from pyruvate. These results strongly suggest that the Δ*cpn60.1* strain experienced a stronger glycolytic pressure than the WT strain, possibly leading to more accumulation of the upstream triose phosphates. Given that accumulation of triose phosphates was reported to be toxic for mycobacteria (Pethe et al., 2010), we further reason that the observed inhibition of the WT growth by the Δ*cpn60.1* strain could be due to the mutant secretion of these potentially toxic metabolites. Interestingly, the WT strain responded to the excess glycerol in the standard biofilm medium by exhibiting the Crabtree effect (Crabtree, 1929; Kosmachevskaya et al., 2015; Rucker et al., 2015), characterized by respiratory reprogramming, ATP downregulation, and glycolytic enhancement. Such an effect has been described in yeast, mammalian cells, and various bacterial species but, to our knowledge, never before in mycobacteria (Crabtree, 1929; Vemuri et al., 2006; Paczia et al., 2012). Importantly, this effect was defective in the Δ*cpn60.1* strain as this mutant had a compromised ability to downregulate the ATP in response to glycerol. We reason that the Cpn60.1-facilitated ATP downregulation may negatively control the rate of glycerol catabolism since the conversion of glycerol to glycerol-3-phosphate requires ATP. This strategy, in combination with the enhanced glycolysis, could ensure the limitation of glycolytic metabolites including triose phosphates and, thus, contribute to restricted generation of MG. Thus, we propose that the Crabtree effect may benefit organisms by restricting the production of the toxic MG.

**FIGURE 8:**
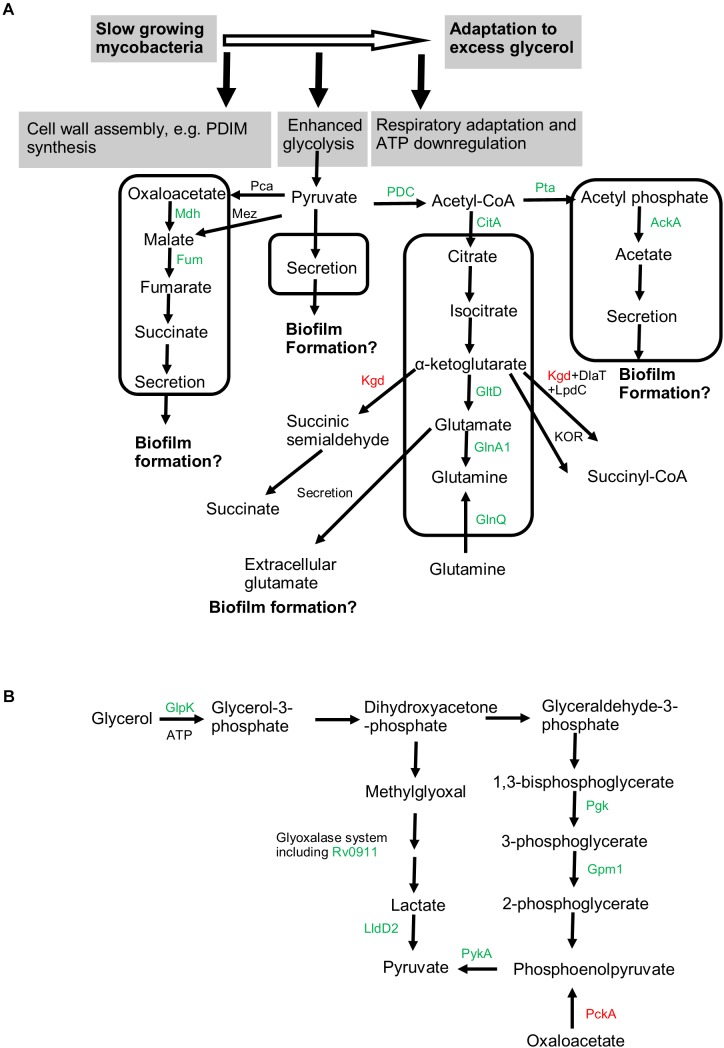
Metabolic pathways associated with adaptation to excess glycerol and mycobacterial biofilm formation. **(A)** Slow-growing mycobacteria respond to excess glycerol by reprogramming the respiratory chain, downregulating ATP production, and boosting the glycolysis [see **(B)** for details]. The pyruvate is proposed to be assimilated into at least four pathways (indicated by box). Proteomics-revealed enzymes participating these pathways were colored (green for upregulation and red for downregulation under excess glycerol). The uptake of glutamine by GlnQ is proposed to occur in the presence of extracellular glutamine. The activation of these pyruvate-assimilating pathways not only prevents the overaccumulation of pyruvate (and thus the toxic methylglyoxal) but also may promote mycobacterial biofilm growth. **(B)** Both the glycolytic pathway and methylglyoxal detoxification pathway are enhanced in the presence of excess glycerol, leading to enhanced production of the end product pyruvate. Related proteins identified by proteomic analysis were indicated and colored. In particular, PckA, catalyzing predominantly the gluconeogenesis, was downregulated. Mdh, malate dehydrogenase; Fum, fumarate hydratase; Pca, pyruvate carboxylase; Mez, malic enzyme; PDC, pyruvate dehydrogenase complex; CitA, citrate synthase II; Kgd, alpha-ketoglutarate decarboxylase; DlaT, dihydrolipoamide acyltransferase; LpdC, dihydrolipoamide dehydrogenase; GltD, glutamate synthase (small subunit); GlnA1, glutamine synthetase; GlnQ, probable glutamine transporter; Pta, phosphate acetyl-transferase; AckA, acetate kinase; Glpk, glycerol kinase; Pgk, phosphoglycerate kinase; Gpm1, phosphoglycerate mutase 1; PykA, pyruvate kinase; Rv0911, putative glyoxalase; LldD2, lactate dehydrogenase; PckA, phosphoenolpyruvate carboxykinase.

The Crabtree effect is partially fulfilled by secretion of metabolites such as lactate and acetate to limit accumulation of pyruvate (Paczia et al., 2012; Sokolov et al., 2015). Mycobacteria are believed to produce marginal lactate from pyruvate (Billig et al., 2017). It is known that acetyl-CoA can be converted to acetate *via* the Pta–AckA pathway (Figure 8A; Rucker et al., 2015). Pta and AckA are encoded in a putative operon *rv0407*-*rv0409* in mycobacteria. Interestingly, both the *rv0407*-encoded Fgd1 and *rv0408*-encoded Pta were upregulated, suggesting the activation of the Pta–AckA pathway under excess glycerol and during biofilm formation. Therefore, pyruvate may be partially directed to acetate *via* acetyl-CoA (Figure 8A). Indeed, acetate was found in the culture filtrates. Since acetic acid was shown to act as a biofilm-stimulating volatile metabolite in other bacteria (Chen et al., 2015), we propose that the acetate flux may contribute to mycobacterial biofilm formation (Figure 8A).

The acetyl-CoA may also be assimilated into the oxidative TCA (Figure 8A). We observed upregulation of fumarate hydratase, malate dehydrogenase, and citrate synthase, pointing to enhancement of TCA under excess glycerol. However, as the α-ketoglutarate decarboxylase (Kgd) (Tian et al., 2005) was downregulated and the alternative α-ketoglutarate ferredoxin oxidoreductase (KOR) was proposed to function predominantly when mycobacteria feed on fatty acids (Baughn et al., 2009), it is likely that the α-ketoglutarate oxidation is not favored under excess glycerol (Figure 8A). Thus, the bacilli may employ reductive and oxidative TCA simultaneously, i.e., from oxaloacetate (or malate) to succinate (the reductive branch) and from citrate to α-ketoglutarate (the oxidative branch) (Figure 8A). If this is the case, succinate, the end product of the reductive TCA, may be secreted, a phenomenon observed for other scenarios such as hypoxia (Watanabe et al., 2011; Eoh and Rhee, 2013). Indeed, succinate secretion was observed under excess glycerol. In addition to pyruvate assimilation, the succinate flux may facilitate mycobacterial adaptation and biofilm formation by reoxidizing NADH and by providing essential precursor metabolites.

In addition to these metabolic adaptations, we also observed upregulation of GltD and GlnA1 (Tullius et al., 2003; Viljoen et al., 2013) under excess glycerol and during biofilm growth, suggesting the activation of the glutamate/glutamine pathway (Figure 8A). We reason that this pathway may assimilate the α-ketoglutarate produced in the oxidative TCA (Figure 8A). The activation of this metabolic pathway was confirmed by the secretion of glutamate in both strains. Interestingly, no or only a limited amount of glutamine was secreted, suggesting that the function of this metabolite in mycobacterial biofilm formation, if any, is primarily exerted intracellularly. Considering that disruption of glutamine/glutamate metabolism compromised biofilm growth of *Bacillus subtilis*, *Enterococcus faecalis*, and *Pseudomonas aeruginosa* (Hassanov et al., 2018), we propose that mycobacterial biofilm formation may be linked to the glutamate/glutamine flux (Figure 8A).

In addition, our results suggest that other pathways, in addition to the Crabtree effect, could contribute to mycobacterial adaptation to excess glycerol and biofilm growth. For instance, methylation might facilitate mycobacterial biofilm growth and/or bacterial fitness under excess glycerol as suggested by the upregulation of 10 (putative) methyltransferases. The involvement of methylation in bacterial biofilm formation was reported in *Campylobacter jejuni*. In this organism, 2′-*O*-methylation of 23S rRNA was required for optimal biofilm formation (Salamaszynska-Guz et al., 2017). In addition, DNA methylation in *Salmonella enterica* serovar Enteritidis was shown to contribute to the biofilm formation of these bacteria by affecting the production of biofilm extracellular compounds (Aya Castaneda Mdel et al., 2015). Based on these observations, we propose that methylation-mediated epigenetic modifications could play important roles in mycobacterial biofilm formation. In addition to this, our proteomic analysis also suggested that iron metabolism could play some roles in mycobacterial biofilm formation. In mycobacteria, the ESX-3 type VII secretion system is required for iron uptake (Serafini et al., 2013; Tufariello et al., 2016). A component of this system [e.g., EccA3 encoded in the *esx-3* locus (Tufariello et al., 2016)] was enhanced during optimal biofilm condition. This observation, together with the downregulation of IdeR and upregulation of BfrA, strongly points to an enhanced iron uptake during mycobacterial biofilm growth. This notion is supported by a previous study in *M. smegmatis* showing the requirement of iron uptake for biofilm formation (Ojha and Hatfull, 2007). Very recently, Rizzi et al. (2019) demonstrated a close link between *Bacillus subtilis* biofilm formation and the iron acquisition and proposed that the biofilm matrix could enhance the efficacy of iron-transporting siderophores. How iron metabolism affects bacterial biofilm remains less understood. The concomitant upregulation of heme-producing proteins as suggested by our proteomic analysis suggests that the intracellular iron pool may be directed, at least partially, to heme biosynthesis during biofilm formation. Interestingly, heme was proposed to function as a signaling molecule that modulates biofilm formation in *Bacillus cereus* (Hussain et al., 2018).

The present work raises an interesting question as to how Cpn60.1 participated in the glycerol-induced ATP downregulation. This is particularly puzzling since proteomic analysis did not reveal significant differences in terms of respiratory protein levels between the WT and Δ*cpn60.1* strains. A potential explanation could be linked to the defective PDIM/PGL production in the Δ*cpn60.1* strain. This may lead to constant cell envelope stress, which was reported to correlate with enhanced ATP production (Shetty and Dick, 2018). Indeed, the isoniazid inductible proteins (IniA/C), which are associated with cell envelope stress (Boot et al., 2017), were upregulated upon Cpn60.1 loss (Supplementary Table S2). However, the PMM50 strain (deficient for PDIM/PGL) was not defective in the glycerol-triggered ATP downregulation (data not shown), ruling out this explanation. Another interesting question is that how the metabolites identified in this study were secreted by mycobacteria. Indeed, understanding the secretion pathways of these metabolites may provide some interesting targets that can be further interfered to counteract mycobacterial biofilm formation. However, the transport of these metabolites is currently poorly understood in mycobacteria. In the case of succinate, mycobacteria encode DctA (Rv2443), a putative transporter of C_4_-dicarboxylates including succinate (Fang et al., 2012). The DctA protein in *Corynebacterium glutamicum* was demonstrated to uptake TCA intermediates (e.g., succinate) dependently of proton motive force (Youn et al., 2009). Whether DctA was responsible for succinate export during mycobacterial biofilm formation remains to be investigated.

This study has some limitations. For instance, the proposed metabolic adaptations (e.g., the glutamate/glutamine pathway), as supported by our proteomic analysis and the quantification of terminal metabolites, could be corroborated by metabolomics and determination of the activities of specific enzymes in those pathways.

Our findings have important implications. The metabolic pathways identified here, including the secretion of several metabolites, may be further explored to obtain potential biomarkers for the detection of mycobacterial biofilms. Indeed, many nontuberculous mycobacteria such as *Mycobacterium chelonae* are often found in biofilms formed in domestic water distribution system, which may represent an important source for human infection (Chakraborty and Kumar, 2019). Therefore, the development of proper methods that can be routinely applied for the detection of mycobacterial biofilm residing in water supply system is necessary. In addition to mycobacteria, biofilms formed by other bacteria such as *Bacillus subtilis* exhibited similar metabolite secretion (i.e., acetate) (Chen et al., 2015), suggesting that the biofilm-associated metabolic pathways identified in this study may represent a more general metabolic adaptations required for biofilm formation in bacteria. In the food industry, biofilm formation by organisms including *Bacillus cereus* is a difficult-to-avoid issue, leading to food spoilage and, consequently, huge economic loss and foodborne infectious diseases (Majed et al., 2016; Hussain et al., 2018). Based on the findings of the present study, we propose that pyruvate, acetate, succinate, and/or glutamate may be further investigated to test their potential value as biofilm biomarkers for, e.g., home water distribution system and food industry.

In summary, we identified that mycobacterial biofilm formation is associated with adaptation to excess glycerol and the concomitant establishment of the Crabtree effect characterized by respiratory adaptation, energetic (ATP) downregulation, glycolytic enhancement, and secretion of pyruvate, succinate, acetate, and glutamate (Figure 8A). These metabolites may be considered as valuable biofilm markers. Cpn60.1 participates in this adaptation by facilitating the ATP downregulation and controlling these metabolic adaptations, thereby restricting overabundance of pyruvate and the upstream MG (Figure 8B). Due to the problematic Crabtree effect, the Δ*cpn60.1* strain is more susceptible to the excess glycerol and suffers from MG-associated growth defect, resulting in its biofilm failure under the standard biofilm medium.

## Author Contributions

SZ and VF designed the experiments and wrote the manuscript with contributions from all co-authors. SZ performed the experiments and analyzed the data. SZ, PC, and MD performed the lipid analysis. SZ, PL, and RW performed the proteomic analysis. SZ, DY, RW, and VF analyzed the proteomic data. AB and VF corrected the manuscript and provided necessary funds.

## Conflict of Interest Statement

The authors declare that the research was conducted in the absence of any commercial or financial relationships that could be construed as a potential conflict of interest.

## Footnotes

1 https://mycobrowser.epfl.ch/

2 https://www.ncbi.nlm.nih.gov/Structure/cdd/wrpsb.cgi

3 https://www.uniprot.org/

## Supplementary Material

FIGURE S1 Growth comparison between WT and PMM50 (PDIM^−^/PGL^−^) BCG strains. Washed BCG cultures were subgrown in 6% glycerol Sauton’s medium for 9 days before OD600 measurement. No significant difference by unpaired *t*-test was observed. The experiment was done in triplicate.

FIGURE S2 PDIM and PGL analysis. PDIM **(A)** and PGL **(B)** analysis by TLC of BCG biofilms grown under 4% glycerol Sauton’s medium. Representative TLC plates are shown. **(C)** Relative lipid amount was based on band intensity. The data were pooled from two independent experiments (each in duplicate or triplicate). ^∗∗∗^ and #, *p* < 0.001 and 0.0001, respectively, relative to corresponding WT data by unpaired *t*-test.

TABLE S1 Selected proteins with altered expression under 6% glycerol Sauton’s medium relative to 0.2% glycerol Sauton’s medium.

TABLE S2 Selected proteins with altered expression in Δ*cpn60.1* under 4% glycerol Sauton’s medium.
