# Supplementary figures and images for: Cpn60.1 (GroEL1) Contributes to Mycobacterial Crabtree Effect: Implications for Biofilm Formation
Source: Front Microbiol. 2019 Jun 11;10:1149. doi: 10.3389/fmicb.2019.01149 (PMC6579834; doi:10.3389/fmicb.2019.01149)

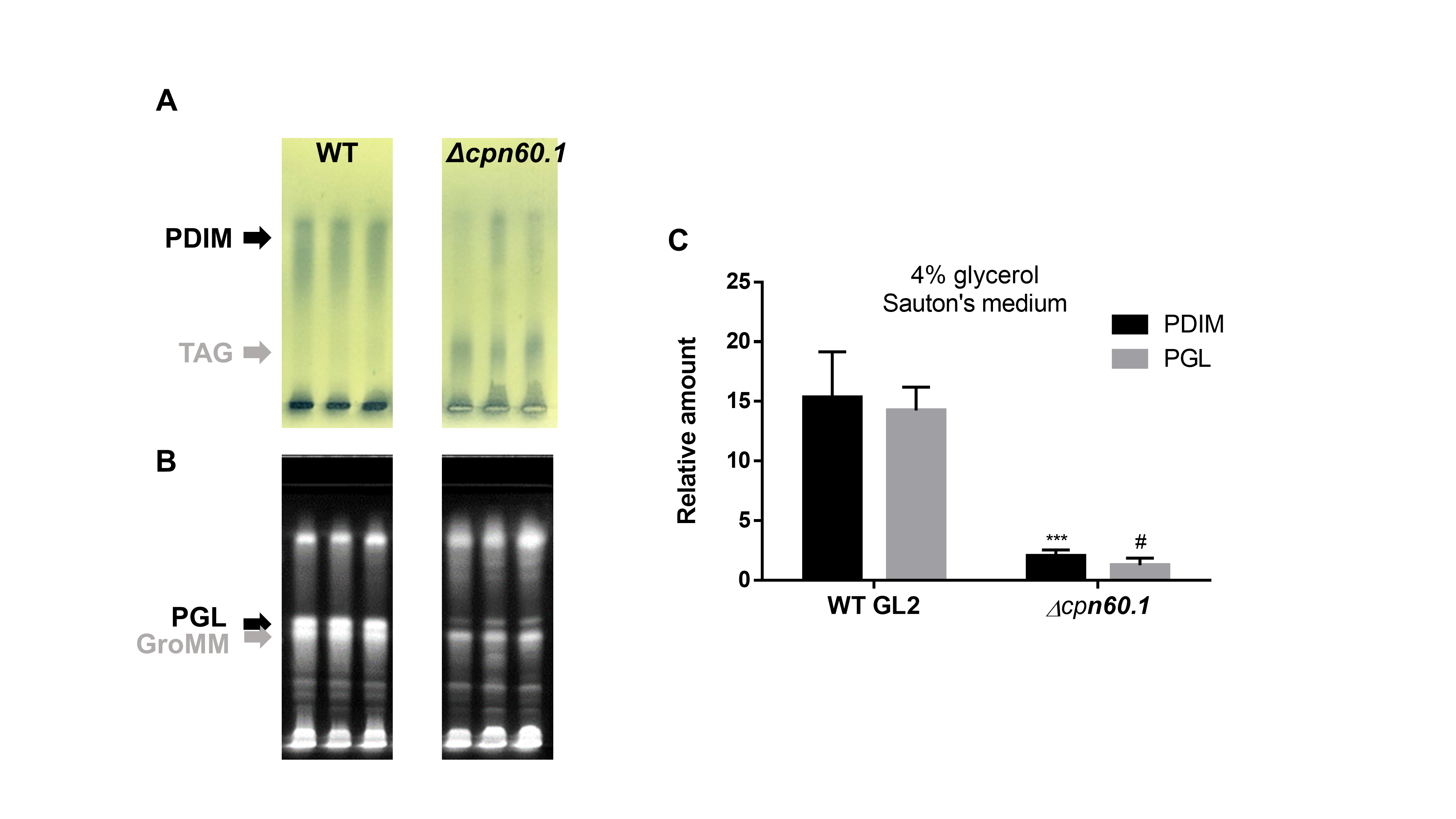

Supplement: FIGURE S2 — PDIM and PGL analysis. PDIM (A) and PGL (B) analysis by TLC of BCG biofilms grown under 4% glycerol Sauton’s medium. Representative TLC plates are shown. (C) Relative lipid amount was based on band intensity. The data were pooled from two independent experiments (each in duplicate or triplicate). ∗∗∗ and #, p < 0.001 and 0.0001, respectively, relative to corresponding WT data by unpaired t-test. [file Image_2.TIF]
