# Supplementary material for: Cpn60.1 (GroEL1) Contributes to Mycobacterial Crabtree Effect: Implications for Biofilm Formation
Source: Front Microbiol. 2019 Jun 11;10:1149. doi: 10.3389/fmicb.2019.01149 (PMC6579834; doi:10.3389/fmicb.2019.01149)
Supplement: TABLE S1 — Selected proteins with altered expression under 6% glycerol Sauton’s medium relative to 0.2% glycerol Sauton’s medium. [file Table_1.DOCX]

**Proteomic procedure**

**Protein extraction**

The protein preparation for the differential label-free proteomic analyses was performed as described previously (Deschoenmaeker et al., 2017). The biomass was treated with a lysis buffer (6 M guanidine HCl, 50 mM K_2_HPO_4_/KH_2_PO_4_, pH 8.5) followed by ultrasonication (3х10 s, 20% amplitude; U50 IKA Technik). 50 mg of extracted proteins were reduced and alkylated. The proteins were recovered by acetone precipitation and digested with 0.005% (w/v) trypsin (Promega V5111) in 25 mM (w/v) NH_4_HCO_3_ (pH 8.5). The trypsin treatment was stopped by adding 0.1% formic acid (v/v).

**Separation of peptides**

Prior to mass spectrometry (MS) analysis, reverse phase chromatography was used to separate the extracts. The reverse-phase column (length 15 cm, diameter 75 mm, flow 300 nl/min; PepMap C18, Dionex) was equilibrated with 4% (v/v) acetonitrile for 20 min and peptide elution was carried out over an acetonitrile gradient from 4% to 35% (v/v) for 120 min. The separated peptides were then analyzed online by TripleTOF 5600 mass spectrometer (AB Sciex, USA).

**SWATH acquisition**

Peptide spectra were acquired in a data-dependent (DDA) and data-independent (DIA) acquisitions modes. The MS/MS library was acquired in the DDA mode and analyzed by ProteinPilot soft-ware (version 4.5, AB Sciex, USA) using the algorithm Paragon (version 4.5.0.0, AB Sciex, USA). Briefly, the trypsin was chosen as the cleavage specificity and alkylation (C) set to iodoacetamide, carbamidomethylation as fixed modifications, oxidation (M) and deamination (N, Q) as variable modifications were set. All biological modifications and amino acid substitutions were considered and a thorough ID search was applied with a peptide confidence set at 0.99. The raw spectral data obtained served as the input for ProteinPilot against the Mycobacterium bovis Uniprot database. For the database search, the cut-off peptide confidence limit was set at 95%. ProteinPilot provided a global false discovery rate of 1% and a local false discovery rate of 5%. Accumulation time was set to 0.1 s for MS1 scan and 65 ms for MS2 scan, with total cycle time being approximately 3.8 s. For the SWATH analysis (DIA, AB Sciex), 32 incremental steps defined as windows of 25 m/z containing 1 m/z for the overlap of window was passed over the full mass range (400–1250 m/z).

Peak intensity method was used for the quantitation of peptides. The ion chromatogram of top six fragmented peptides was extracted, and their area was integrated over the 15 min on six transitions. The tolerance was set at 100 ppm. The SWATH data were processed with PeakView software (version 2.1.0.11041, AB Sciex, USA). The retention time (RT) was calculated manually from a group of 15 selected peptides with RT in the range of 20-100 min. Software MarkerView (version 1.2.1, AB Sciex, USA) was used for analysis of the relative abundance of the peptides and statistical analysis. For all experiments, proteins identified with one peptide were rejected for interpretation.

**Data analysis**

Protein hits with a p-value ˂0.05 and a fold change ˂0.8 or ˃1.2 were further analyzed. Protein function classifications were based on Mycobrowser (https://mycobrowser.epfl.ch/), NCBI Conserved Domains search tool (https://www.ncbi.nlm.nih.gov/Structure/cdd/wrpsb.cgi) and UniProt (https://www.uniprot.org/).

**Reference**

Deschoenmaeker, F., Bayon-Vicente, G., Sachdeva, N., Depraetere, O., Cabrera Pino, J.C., Leroy, B., et al. (2017). Impact of different nitrogen sources on the growth of Arthrospira sp. PCC 8005 under batch and continuous cultivation - A biochemical, transcriptomic and proteomic profile. *Bioresour Technol* 237**,** 78-88. doi: 10.1016/j.biortech.2017.03.145.
