# Supplementary material for: Cpn60.1 (GroEL1) Contributes to Mycobacterial Crabtree Effect: Implications for Biofilm Formation
Source: Front Microbiol. 2019 Jun 11;10:1149. doi: 10.3389/fmicb.2019.01149 (PMC6579834; doi:10.3389/fmicb.2019.01149)
Supplement: TABLE S2 — Selected proteins with altered expression in Δcpn60.1 under 4% glycerol Sauton’s medium. [file Table_2.DOCX]

| **BCG Pasteur locus** | **H37Rv locus** | **Description** | **Symbol** | **6% glycerol versus 0.2% glycerol** | |
| --- | --- | --- | --- | --- | --- |
|  |  |  |  | P-value | Fold change |
| Methylation |  |  |  |  |  |
| BCG_0883 | Rv0830 | S-adenosylmethionine-dependent methyltransferase | NA | 0.0005 | 2.164464883 |
| BCG_1741c | Rv1703c | Probable catechol-O-methyltransferase | NA | 0.00213 | 1.694124772 |
| BCG_1067 | Rv1010 | rRNA small subunit methyltransferase A | KsgA | 0.00237 | 2.191819171 |
| BCG_0321 | Rv0281 | S-adenosylmethionine-dependent methyltransferase | NA | 0.00446 | 1.60032137 |
| BCG_1935c | Rv1896c | S-adenosyl-L-methionine-dependent methyltransferase | NA | 0.00726 | 4.673976131 |
| BCG_0781c | Rv0731c | S-adenosylmethionine-dependent methyltransferase | NA | 0.00758 | 1.511072988 |
| BCG_1453 | Rv1392 | S-adenosylmethionine synthetase | MetK | 0.0103 | 0.670352879 |
| BCG_2980c | Rv2959c | rhamnosyl O-methyltransferase | NA | 0.0253 | 2.598822468 |
| BCG_0776c | Rv0726c | S-adenosylmethionine-dependent methyltransferase | NA | 0.03615 | 2.973347292 |
| BCG_3469 | Rv3399 | S-adenosylmethionine-dependent methyltransferase | NA | 0.04775 | 3.302157646 |
| BCG_2976c | Rv2955c | Function unknown but harboring conserved domain indicating a methyltransferase | NA | 0.048 | 1.616587597 |
| PDIM/PGL biosynthesis |  |  |  |  |  |
| BCG_2957 | Rv2935 | Phthiocerol synthesis polyketide synthase type I | PpsE | 0.00012 | 2.896915106 |
| BCG_2973 | Rv2952 | Phthiotriol/phenolphthiotriol dimycocerosates methyltransferase (SAM-dependent) | NA | 0.00144 | 1.704461192 |
| BCG_2971c | Rv2950c | Long-chain-fatty-acid-AMP ligase | FadD29 | 0.00574 | 4.286579468 |
| BCG_2963 | Rv2941 | Fatty-acid-AMP ligase | FadD28 | 0.02054 | 0.660767564 |
| BCG_2961 | Rv2939 | phthiocerol/phthiodiolone dimycocerosyl transferase | PapA5 | 0.02336 | 3.060967137 |
| BCG_2956 | Rv2934 | phthiocerol synthesis polyketide synthase type I | PpsD | 0.03703 | 1.505781558 |
| Lipid metabolism |  |  |  |  |  |
| BCG_0131 | Rv0098 | Fatty acyl CoA thioesterase type III | FcoT | 2.48E-05 | 3.240672657 |
| BCG_3780 | Rv3720 | Fatty acid synthase with domains similar to mycolic acid cyclopropane synthetase and methyltransferase | NA | 5.63E-05 | 1.915854719 |
| BCG_0121 | Rv0088 | Polyketide cyclase/dehydrase | NA | 0.00044 | 2.061608415 |
| BCG_0280c | Rv0242c | 3-oxoacyl-ACP reductase involved in fatty acid biosynthesis (first reduction step) | FabG4 | 0.00046 | 0.505296304 |
| BCG_1602 | Rv1550 | Fatty-acid-CoA ligase | FadD11 | 0.00052 | 3.994528009 |
| BCG_0546c | Rv0503c | Cyclopropane mycolic acid synthase | CmaA2 | 0.00065 | 0.459133234 |
| BCG_3905c | Rv3842c | glycerophosphoryl diester phosphodiesterase | GlpQ1 | 0.00083 | 8.764885017 |
| BCG_0132 | Rv0099 | fatty-acid-CoA ligase | FadD10 | 0.00187 | 3.878212427 |
| BCG_2502c | Rv2484c | Diacyglycerol O-acyltransferase (triacylglycerol synthase) | NA | 0.00203 | 2.821486038 |
| BCG_1579c | Rv1527c | Polyketide synthase | Pks5 | 0.00206 | 0.312707814 |
| BCG_2529 | Rv2509 | Short-chain type dehydrogenase/reductase; involved in reduction of β-keto mycolate? | NA | 0.00212 | 1.623826034 |
| BCG_0679c | Rv0632c | Enoyl-CoA hydratase involved in lipid degradation | EchA3 | 0.00238 | 2.464378703 |
| BCG_1154 | Rv1094 | Acyl-ACP desaturase | DesA2 | 0.00238 | 0.531844859 |
| BCG_1305c | Rv1245c | short-chain type dehydrogenase/reductase | NA | 0.0028 | 0.562436479 |
| BCG_1923c | Rv1886c | Diacylglycerol acyltransferase/mycolyltransferase; involved in cell wall mycoloylation, such as TDM formation | Ag85B (FbpB) | 0.00314 | 10.77017416 |
| BCG_3086c | Rv3061c | acyl-CoA dehydrogenase | FadE22 | 0.00458 | 0.584967959 |
| BCG_0281 | Rv0243 | 3-ketoacyl-CoA thiolase involved in lipid degradation | FadA2 | 0.00465 | 0.402053705 |
| BCG_2261 | Rv2244 | meromycolate extension acyl carrier protein | AcpM | 0.00676 | 2.098651487 |
| BCG_3162 | Rv3139 | acyl-CoA dehydrogenase involved in lipid degradation | FadE24 | 0.00754 | 0.573254973 |
| BCG_1129c | Rv1071c | enoyl-CoA hydratase involved in lipid degradation | EchA9 | 0.00771 | 0.702207775 |
| BCG_2262 | Rv2245 | 3-oxoacyl-ACP synthase 1 | KasA | 0.00828 | 1.713733703 |
| BCG_1717 | Rv1679 | acyl-CoA dehydrogenase | FadE16 | 0.00877 | 2.405520096 |
| BCG_3876c | Rv3814c | 1-acyl-sn-glycerol-3-phosphate acyltransferase | NA | 0.00974 | 0.377206379 |
| BCG_0925 | Rv0873 | acyl-CoA dehydrogenase | FadE10 | 0.01353 | 0.5400607 |
| BCG_1789c | Rv1750c | fatty-acid-CoA ligase involved in lipid degradation | FadD1 | 0.01457 | 2.60607284 |
| BCG_1901c | Rv1865c | short-chain type dehydrogenase | NA | 0.01491 | 0.600890109 |
| BCG_3599c | Rv3535c | acetaldehyde dehydrogenase thought to be involved in lipid degradation | HsaG | 0.01528 | 0.595737641 |
| BCG_0684 | Rv0635 | (3R)-hydroxyacyl-ACP dehydratase subunit | HadA | 0.01623 | 2.032745999 |
| BCG_1546 | Rv1484 | NADH-dependent enoyl-[ACP] reductase | InhA | 0.01632 | 2.788945468 |
| BCG_0685 | Rv0636 | (3R)-hydroxyacyl-ACP dehydratase subunit | HadB | 0.01731 | 3.071637603 |
| BCG_0877c | Rv0824c | acyl-ACP desaturase, catalyzing the conversion of saturated fatty acids to unsaturated fatty acids | DesA 1 | 0.01734 | 0.459251194 |
| BCG_2263 | Rv2246 | 3-oxoacyl-ACP synthase 2 | KasB | 0.01871 | 1.561446965 |
| BCG_2968c | Rv2946c | polyketide synthase | Pks1 | 0.02029 | 2.472378688 |
| BCG_0957 | Rv0905 | enoyl-CoA hydratase | EchA6 | 0.02213 | 2.441121708 |
| BCG_2783c | Rv2766c | NAD(P)-dependent oxidoreductase; 3-ketoacyl-ACP reductase? | NA | 0.02577 | 3.076132934 |
| BCG_0153 | Rv0119 | fatty-acid-CoA ligase | FadD7 | 0.02858 | 2.393634502 |
| BCG_2613 | Rv2590 | fatty-acid-CoA ligase | FadD9 | 0.03077 | 0.224554051 |
| BCG_0357c | Rv0317c | glycerophosphoryl diester phosphodiesterase | GlpQ2 | 0.03434 | 2.884201947 |
| BCG_2260 | Rv2243 | malonyl CoA-acyl carrier protein transacylase | FabD | 0.03599 | 0.538857034 |
| BCG_1656 | Rv1618 | acyl-CoA thioesterase II involved in lipid metabolism | TesB1 | 0.03986 | 1.848439588 |
| BCG_1489c | Rv1428c | Possible glycerol acyltransferase | NA | 0.04201 | 0.716579071 |
| BCG_1075c | Rv1018c | bifunctional N-acetylglucosamine-1-phosphate uridyltransferase/glucosamine-1-phosphate acetyltransferase; involved in synthesis of UDP-N-acetylglucosamine (UDP-GlcNAc); involved in peptidoglycan and lipopolysaccharide biosynthesis | GlmU | 0.0473 | 0.426862648 |
| BCG_2495c | Rv2475c | acyl-CoA thioesterase | NA | 0.04827 | 1.503591279 |
| BCG_0686 | Rv0637 | (3R)-hydroxyacyl-ACP dehydratase subunit | HadC | 0.04892 | 1.745573173 |
| BCG_0279c | Rv0241c | 3-hydroxyacyl-thioester dehydratase; possible component of fatty acid synthesis type II | HtdX | 0.049 | 0.452193707 |
| DosR regulon |  |  |  |  |  |
| BCG_2650 | Rv2623 | universal stress protein | TB31.7 | 0.00567 | 0.566682115 |
| BCG_2013 | Rv1996 | universal stress protein | NA | 0.00936 | 0.424766065 |
| BCG_3153c | Rv3130c | diacyglycerol O-acyltransferase (triacylglycerol synthase) | Tgs1 | 0.01087 | 0.478316023 |
| BCG_3154 | Rv3131 | NAD(P)H nitroreductase | NA | 0.01479 | 0.388665277 |
| BCG_2023 | Rv2006 | trehalose-6-phosphate phosphatase involved in trehalose biosynthesis | OtsB1 | 0.02073 | 0.381405087 |
| BCG_2022c | Rv2005c | universal stress protein | NA | 0.03102 | 0.540161522 |
| BCG_2047c | Rv2028c | universal stress protein | NA | 0.03111 | 0.372049944 |
| BCG_2049c | Rv2030c | function unknown | NA | 0.04575 | 0.376204934 |
| BCG_0113 | Rv0080 | pyridoxamine 5'-phosphate oxidase family protein | NA | 0.04636 | 0.252250356 |
| BCG_2653c | Rv2626c | hypoxic response protein | Hrp1 | 0.05008 | 0.528720963 |
| Stress tolerance, detoxification and repair |  |  |  |  |  |
| BCG_0963 | Rv0911 | With conserved domain similar to *Streptomyces griseus* SgaA, which suppresses the growth disturbances caused by high osmolarity; putative glyoxalase I (lactoylglutathione lyase) | NA | 3.81E-06 | 22.43998622 |
| BCG_2900c | Rv2878c | Secreted protein; possible protein disulfide oxidoreductase; with thioredoxin like domain; might contribute to oxidative stress | Mpt53 | 0.0002 | 4.546534278 |
| BCG_1971 | Rv1932 | 2-Cys peroxiredoxin; thiol peroxidase; defense against oxidative stress by detoxifying peroxides | Tpx | 0.00031 | 1.925206172 |
| BCG_3732c | Rv3674c | Endonuclease III; DNA repair enzyme | Nth | 0.00102 | 0.138185347 |
| BCG_3207c | Rv3181c | Antitoxin | VapB45 | 0.00185 | 5.257182459 |
| BCG_3131 | Rv3106 | NADPH-ferredoxin reductase | FprA | 0.00329 | 3.642993765 |
| BCG_2447 | Rv2428 | Alkyl hydroperoxide reductase C protein; constitutes an NADH-dependent peroxidase and peroxynitrite reductase that provides protection against oxidative stress | AhpC | 0.00433 | 1.999617281 |
| BCG_0569 | Rv0526 | thioredoxin; possible protein disulfide reductase; antioxidant function | NA | 0.00652 | 3.745067083 |
| BCG_2875 | Rv2855 | NADPH-dependent mycothione reductase | Mtr | 0.00776 | 1.64112066 |
| BCG_1674 | Rv1636 | iron-regulated universal stress protein | TB15.3 | 0.00903 | 1.761843836 |
| BCG_2754 | Rv2740 | epoxide hydrolase | EphG | 0.01346 | 0.290796174 |
| BCG_3972 | Rv3914 | thioredoxin; protein disulfide oxidoreductase activity | TrxC | 0.01563 | 1.533701845 |
| BCG_2618 | Rv2595 | antitoxin | VapB40 | 0.01679 | 1.333802256 |
| BCG_0019 | Rv3913 | NADPH dependent thioredoxin reductase; defense against oxidative stress | TrxB2 | 0.01919 | 2.092807682 |
| BCG_1185 | Rv1124 | epoxide hydrolase | EphC | 0.0228 | 2.88143514 |
| BCG_2255c | Rv2238c | peroxiredoxin; antioxidant role | AhpE | 0.02419 | 1.894383224 |
| BCG_2997c | Rv2976c | uracil-DNA glycosylase involved in base excision repair; defense against DNA damage | Ung | 0.02938 | 2.438675225 |
| BCG_3256c | Rv3226c | SOS response-associated peptidase | NA | 0.02969 | 2.83734291 |
| BCG_1771c | Rv1732c | thioredoxin family protein with similarity to peroxiredoxins; possible antioxidant role | NA | 0.03786 | 1.594922601 |
| Transcription, translation and amino acid metabolism |  |  |  |  |  |
| BCG_1138c | Rv1080c | Transcription elongation factor | GreA | 0.00024 | 0.414970798 |
| BCG_2237 | Rv2220 | Glutamine synthetase | GlnA1 | 0.00026 | 2.18457663 |
| BCG_1588 | Rv1536 | isoleucine--tRNA ligase | IleS | 0.00029 | 0.322850387 |
| BCG_0716 | Rv0667 | DNA-directed RNA polymerase (beta chain) | RpoB | 0.00038 | 0.630486207 |
| BCG_3770 | Rv3710 | 2-isopropylmalate synthase involved in leucine synthesis at first step | LeuA | 0.00047 | 0.432277844 |
| BCG_0226c | Rv0189c | Dihydroxy-acid dehydratase involved in isoleucine and valine synthesis | IlvD | 0.00053 | 2.502118032 |
| BCG_3663c | Rv3598c | Lysine-tRNA ligase | LysS | 0.00068 | 3.105295511 |
| BCG_2074c | Rv2055c | 30S ribosomal protein S18 | RpsR2 | 0.00162 | 6.812078578 |
| BCG_3016c | Rv2995c | 3-isopropylmalate dehydrogenase | LeuB | 0.00225 | 1.443628354 |
| BCG_3648c | Rv3583c | RNA polymerase-binding transcription factor | CarD | 0.00232 | 0.266035173 |
| BCG_0766 | Rv0716 | 50S ribosomal protein L5 | RplE | 0.00271 | 0.7165234 |
| BCG_0688 | Rv0639 | Transcription termination/antitermination protein | NusG | 0.00359 | 0.759361838 |
| BCG_3008c | Rv2987c | Probable 3-isopropylmalate dehydratase (small subunit) participating leucine biosynthesis | LeuD | 0.00435 | 0.562591037 |
| BCG_3921c | Rv3858c | NADH-dependent glutamate synthase small subunit | GltD | 0.00469 | 2.651575829 |
| BCG_1354 | Rv1294 | Homoserine dehydrogenase involved in methionine and threonine biosynthesis | ThrA | 0.00513 | 0.711087406 |
| BCG_2925c | Rv2904c | 50S ribosomal protein L19 | RplS | 0.00657 | 3.440853824 |
| BCG_2861c | Rv2841c | Probable N utilization substance protein A involved in both termination and antitermination of transcription | NusA | 0.00709 | 0.766827348 |
| BCG_0428 | Rv0391 | O-succinylhomoserine sulfhydrylase involved in methionine biosynthesis | MetZ | 0.00716 | 1.817082149 |
| BCG_0936c | Rv0884c | phosphoserine aminotransferase involved in serine and pyridoxine 5'-phosphate synthesis | SerC | 0.00952 | 1.645547645 |
| BCG_0717 | Rv0668 | DNA-directed RNA polymerase (beta' chain) | RpoC | 0.01039 | 0.475435077 |
| BCG_1261c | Rv1201c | 2,3,4,5-tetrahydropyridine-2,6-dicarboxylate N-succinyltransferase involved in lysine biosynthesis | DapD | 0.01076 | 1.527378945 |
| BCG_3522c | Rv3457c | Probable DNA-directed RNA polymerase (alpha chain) | RpoA | 0.01087 | 0.647689049 |
| BCG_0771 | Rv0721 | 30S ribosomal protein S5 | RpsE | 0.01441 | 0.731372398 |
| BCG_0910c | Rv0858c | N-succinyldiaminopimelate aminotransferase involved in lysine biosynthesis (succinylase pathway) | DapC | 0.01741 | 6.358144696 |
| BCG_1688 | Rv1649 | phenylalanine--tRNA ligase subunit alpha | PheS | 0.01798 | 4.244009109 |
| BCG_1867 | Rv1832 | glycine dehydrogenase involved in glycine degradation | GcvB | 0.01869 | 0.318765269 |
| BCG_1691 | Rv1652 | N-acetyl-gamma-glutamyl-phoshate reductase involved in arginine biosynthesis | ArgC | 0.02071 | 0.657201102 |
| BCG_1647 | Rv1609 | anthranilate synthase component I involved in tryptophan synthesis | TrpE | 0.02207 | 1.465880156 |
| BCG_2858c | Rv2838c | ribosome-binding factor | RbfA | 0.0221 | 0.57536817 |
| BCG_1249 | Rv1187 | pyrroline-5-carboxylate dehydrogenase involved in proline catabolic process | RocA | 0.0238 | 2.714682979 |
| BCG_2356 | Rv2334 | O-acetylserine sulfhydrylase involved in cysteine biosynthesis | CysK1 | 0.02488 | 0.493485958 |
| BCG_1915 | Rv1878 | glutamine synthetase involved in glutamine synthesis | GlnA3 | 0.02507 | 0.45048904 |
| BCG_1262 | Rv1202 | succinyl-diaminopimelate desuccinylase involved in lysine synthesis | DapE | 0.02924 | 0.710722906 |
| BCG_1227 | Rv1165 | GTP-binding translation elongation factor | TypA | 0.03157 | 4.602678162 |
| BCG_2556c | Rv2534c | elongation factor P | Efp | 0.03236 | 1.508671252 |
| BCG_3743 | Rv3684 | lyase; possible pyridoxal 5'-phosphate (PLP)-dependent cysteine synthase family protein | NA | 0.03306 | 2.262722055 |
| BCG_1694 | Rv1655 | acetylornithine aminotransferase; arginine biosynthesis | ArgD | 0.03498 | 1.568681206 |
| BCG_2859c | Rv2839c | translation initiation factor IF-2 | InfB | 0.03758 | 0.641452896 |
| BCG_1698 | Rv1659 | argininosuccinate lyase involved in arginine synthesis | ArgH | 0.03838 | 0.424311295 |
| BCG_1752 | Rv1713 | GTPase Der associated with translation process | EngA | 0.03852 | 0.428585038 |
| BCG_3769c | Rv3709c | aspartokinase involved in lysine, methionine and threonine synthesis | Ask | 0.04058 | 0.770159066 |
| BCG_2406 | Rv2392 | phosphoadenosine phosphosulfate reductase; cysteine biosynthesis | CysH | 0.0424 | 0.56348864 |
| BCG_0552 | Rv0509 | glutamyl-tRNA reductase | HemA | 0.0437 | 1.455762444 |
| BCG_2227c | Rv2211c | Aminomethyltransferase catalyzing degradation of glycine | GcvT | 0.04549 | 2.049165857 |
| BCG_1643 | Rv1605 | imidazole glycerol phosphate synthase subunit (probable cyclase) involved in histidine biosynthesis pathway | HisF | 0.04679 | 2.465271143 |
| BCG_2865c | Rv2845c | proline--tRNA ligase | ProS | 0.04688 | 2.20621099 |
| BCG_0289c | Rv0251c | heat shock protein; binding to 30S ribosomal subunit; possibly involved in translation at high temperature | Hsp | 0.04945 | 0.134948032 |
| Metabolic process |  |  |  |  |  |
| BCG_2515c | Rv2495c | Branched-chain keto acid dehydrogenase E2 component | BkdC | 0.0001 | 0.494559223 |
| BCG_1872c | Rv1837c | Malate synthase G involved in glyoxylate shunt (second step) | GlcB | 0.00056 | 0.670890514 |
| BCG_3755c | Rv3696c | glycerol kinase | GlpK | 0.00206 | 4.333919933 |
| BCG_0248 | Rv0211 | phosphoenolpyruvate carboxykinase | PckA | 0.00242 | 0.438012616 |
| BCG_0446 | Rv0407 | F420-dependent glucose-6-phosphate dehydrogenase catalyzing first step of pentose phosphate pathway; might contribute to oxidative stress | Fgd1 | 0.00258 | 1.828471509 |
| BCG_1506c | Rv1445c | 6-phosphogluconolactonase involved in pentose phosphate pathway at second step. | DevB | 0.00464 | 13.46928759 |
| BCG_1158c | Rv1098c | fumarate hydratase participating TCA cycle | Fum | 0.0052 | 1.52520645 |
| BCG_1908c | Rv1872c | L-lactate dehydrogenase required for lactate oxidation | LldD2 | 0.0053 | 2.438923436 |
| BCG_1300 | Rv1240 | malate dehydrogenase | Mdh | 0.01249 | 3.191976862 |
| BCG_2258 | Rv2241 | pyruvate dehydrogenase E1 component | AceE | 0.01462 | 1.425091107 |
| BCG_1510c | Rv1449c | transketolase involved in pentose phosphate pathway | Tkt | 0.01942 | 0.696818045 |
| BCG_3861c | Rv3799c | propionyl-CoA carboxylase beta chain 4 (catalyzing first step of methylmalonyl CoA pathway?) | AccD4 | 0.02018 | 1.722938404 |
| BCG_0941c | Rv0889c | Probable citrate synthase II | CitA | 0.0243 | 1.457459566 |
| BCG_1556 | Rv1493 | Probable methylmalonyl-CoA mutase large subunit | MutB | 0.02781 | 0.437186259 |
| BCG_2517c | Rv2497c | Probable branched-chain keto acid dehydrogenase E1 component, alpha subunit | BkdA | 0.02809 | 0.558839974 |
| BCG_0530 | Rv0489 | 2,3-bisphosphoglycerate-dependent phosphoglycerate mutase involved in glycolysis | Gpm1 | 0.03 | 2.05366062 |
| BCG_0447 | Rv0408 | phosphate acetyltransferase involved in the interconversion of acetate to acetyl-CoA | Pta | 0.03318 | 1.538607109 |
| BCG_2521c | Rv2501c | Acetyl-/propionyl-CoA carboxylase subunit alpha | AccA1 | 0.03436 | 0.623965586 |
| BCG_3093c | Rv3068c | phosphoglucomutase involved in both breakdown and generation of glucose | PgmA | 0.03534 | 2.80739314 |
| BCG_1308c | Rv1248c | multifunctional 2-oxoglutarate dehydrogenase E1 component /2-oxoglutarate dehydrogenase dihydrolipoyllysine-residue succinyltransferase | Kgd | 0.03771 | 0.565399931 |
| BCG_2516c | Rv2496c | Probable branched-chain keto acid dehydrogenase E1 component (beta subunit) involved in degradation of branched chain amino acids | BkdB | 0.03775 | 0.434461075 |
| BCG_1508c | Rv1447c | glucose-6-phosphate 1-dehydrogenase | Zwf2 | 0.03887 | 0.637813977 |
| BCG_1498 | Rv1437 | phosphoglycerate kinase involved in glycolysis | Pgk | 0.0391 | 1.489492043 |
| BCG_1555 | Rv1492 | methylmalonyl-CoA mutase small subunit | MutA | 0.04136 | 0.315177858 |
| BCG_1655 | Rv1617 | pyruvate kinase catalyzing the final step of glycolysis | PykA | 0.04341 | 1.557515339 |
| Regulator and sensor kinase |  |  |  |  |  |
| BCG_0505c | Rv0465c | HTH-type transcriptional regulator | NA | 0.00057 | 0.549161843 |
| BCG_2190c | Rv2175c | DNA-binding regulatory protein | NA | 0.00312 | 7.62274152 |
| BCG_1758 | Rv1719 | IclR family transcriptional regulator | NA | 0.00479 | 3.6672373 |
| BCG_2724 | Rv2711 | iron-dependent repressor and activator | IdeR | 0.00578 | 0.614686939 |
| BCG_2908 | Rv2887 | HTH-type transcriptional regulator | NA | 0.0085 | 2.148847603 |
| BCG_1037 | Rv0982 | Two component sensor kinase | MprB | 0.01102 | 0.374707926 |
| BCG_3912 | Rv3849 | ESX-1 transcriptional regulatory protein | EspR | 0.01231 | 1.532080774 |
| BCG_3010 | Rv2989 | transcriptional regulator | NA | 0.0124 | 0.516636431 |
| BCG_0298c | Rv0260c | transcriptional regulator; uroporphyrinogen-III synthase involved in heme synthesis | NA | 0.01802 | 2.992892787 |
| BCG_1484 | Rv1423 | transcriptional regulator | WhiA | 0.02428 | 2.466565869 |
| BCG_0878c | Rv0825c | TetR/AcrR family transcriptional regulator | NA | 0.03373 | 0.304572077 |
| BCG_2940c | Rv2919c | nitrogen regulatory protein P-II | GlnB | 0.03484 | 2.847081685 |
| BCG_3275c | Rv3246c | two component DNA-binding response regulator | MtrA | 0.04318 | 0.528760418 |
| BCG_3330c | Rv3301c | phosphate transport system transcriptional regulator; regulation of phosphate uptake | PhoY | 0.04506 | 1.864228401 |
| Transporter |  |  |  |  |  |
| BCG_3299 | Rv3270 | Manganese/zinc-exporting P-type ATPase | CtpC | 3.92E-05 | 0.131295997 |
| BCG_0322 | Rv0282 | ESX-3 secretion system protein | EccA3 | 0.00383 | 1.688408824 |
| BCG_1826 | Rv1794 | ESX secretion-associated protein EspG | NA | 0.0079 | 3.332547642 |
| BCG_3225c | Rv3200c | transmembrane cation transporter | NA | 0.00913 | 0.570231618 |
| BCG_3936c | Rv3881c | Secreted ESX-1 substrate protein B | EspB | 0.01004 | 0.663570243 |
| BCG_0243c | Rv0206c | transmembrane transport protein; trehalose monomycolate transporter | MmpL3 | 0.01114 | 0.47527858 |
| BCG_3739 | Rv3680 | anion transporter ATPase | NA | 0.01122 | 0.588256168 |
| BCG_0704 | Rv0655 | Possible ribonucleotide-transport ATP-binding protein ABC transporter | Mkl | 0.0136 | 1.681189152 |
| BCG_3945c | Rv3889c | ESX-2 secretion-associated protein | EspG2 | 0.01473 | 3.974563417 |
| BCG_1815 | Rv1782 | ESX-5 type VII secretion system protein | EccB5 | 0.01926 | 2.059967538 |
| BCG_1816 | Rv1783 | ESX-5 type VII secretion system protein | EccC5 | 0.01939 | 2.32937636 |
| BCG_2610c | Rv2587c | protein translocase subunit | SecD | 0.0223 | 0.371355134 |
| BCG_0489c | Rv0450c | transmembrane transport protein | MmpL4 | 0.03153 | 0.33662097 |
| BCG_2586 | Rv2564 | glutamine ABC transporter ATP-binding protein | GlnQ | 0.03784 | 2.361407707 |
| BCG_1830 | Rv1798 | ESX-5 type VII secretion system protein | EccA5 | 0.03921 | 2.695777529 |
| BCG_3941c | Rv3886c | membrane-anchored mycosin; type VII secretion system ESX-2 serine protease mycosin | MycP2 | 0.04101 | 2.766689947 |
| BCG_2114c | Rv2094c | Sec-independent protein translocase membrane-bound protein; twin-arginine translocation system | TatA | 0.04183 | 0.168587185 |
| BCG_1829 | Rv1797 | ESX-5 type VII secretion system protein EccE | EccE5 | 0.04383 | 5.638122932 |
| BCG_2705 | Rv2692 | TRK system potassium uptake protein | CeoC | 0.04513 | 2.344991733 |
| BCG_2701c | Rv2688c | antibiotic ABC transporter ATP-binding protein | NA | 0.04588 | 0.333826224 |
| BCG_1298 | Rv1238 | sugar ABC transporter ATP-binding protein | SugC | 0.04596 | 1.625636954 |
| Others |  |  |  |  |  |
| BCG_1594c | Rv1542c | Hemoglobin involved in oxygen transport | GlbN | 8.74E-05 | 8.168665362 |
| BCG_0005 | Rv0005 | DNA gyrase (subunit B); ATP-hydrolyzing DNA topoisomerase II | GyrB | 0.00154 | 0.356826127 |
| BCG_0984c | Rv0931c | Serine/threonine-protein kinase | PknD | 0.00155 | 1.473119315 |
| BCG_1523 | Rv1462 | Fe-S cluster assembly protein | NA | 0.00205 | 0.556581602 |
| BCG_3075c | Rv3051c | Ribonucleoside-diphosphate reductase catalyzing synthesis of deoxyribonucleotides and therefore involved in DNA replication | NrdE | 0.0025 | 0.2884691 |
| BCG_1343 | Rv1284 | Beta-carbonic anhydrase | CanA | 0.00277 | 0.383420674 |
| BCG_3704c | Rv3646c | ATP-independent DNA topoisomerase I | TopA | 0.00487 | 2.816065365 |
| BCG_0006 | Rv0006 | DNA gyrase subunit A (ATP hydrolyzing) | GyrA | 0.00574 | 0.403288152 |
| BCG_1912 | Rv1876 | bacterioferritin | BfrA | 0.00628 | 2.088740894 |
| BCG_3319c | Rv3290c | L-lysine-epsilon aminotransferase | Lat | 0.00661 | 0.096881926 |
| BCG_3322 | Rv3293 | piperideine-6-carboxylic acid dehydrogenase involved in L-alpha-aminoadipic acid (L-AAA) biosynthesis (in the second step; the first step is promoted by Lat) | Pcd | 0.0125 | 0.719504565 |
| BCG_1152c | Rv1092c | Probable pantothenate kinase involved in coenzyme A biosynthesis | CoaA | 0.01437 | 1.442207281 |
| BCG_1452 | Rv1391 | DNA/pantothenate metabolism flavoprotein; bifunctional phosphopantothenoylcysteine decarboxylase/phosphopantothenate--cysteine ligase involved in CoA biosynthesis | Dfp | 0.01717 | 0.447227523 |
| BCG_0159 | Rv0125 | serine protease | PepA | 0.0183 | 6.063086818 |
| BCG_0045c | Rv0015c | serine/threonine-protein kinase | PknA | 0.01964 | 0.621267091 |
| BCG_3302 | Rv3273 | transmembrane carbonic anhydrase | NA | 0.02165 | 0.455248244 |
| BCG_3773 | Rv3713 | Possible cobyric acid synthase involved in cobalamin synthesis | CobQ2 | 0.02179 | 3.312708304 |
| BCG_0559c | Rv0516c | anti-anti-sigma factor | NA | 0.02556 | 0.289017749 |
| BCG_2210 | Rv2194 | ubiquinol-cytochrome C reductase cytochrome subunit C, component of the highly energy efficient oxidase branch | QcrC | 0.02914 | 0.076514786 |
