# Supplementary material for: Cpn60.1 (GroEL1) Contributes to Mycobacterial Crabtree Effect: Implications for Biofilm Formation
Source: Front Microbiol. 2019 Jun 11;10:1149. doi: 10.3389/fmicb.2019.01149 (PMC6579834; doi:10.3389/fmicb.2019.01149)
Supplement: Supplementary file 5 [file Table_3.DOCX]

| **BCG Pasteur locus** | **H37Rv locus** | **Description** | **Symbol** | **WT versus *Δcpn60.1*** | |
| --- | --- | --- | --- | --- | --- |
|  |  |  |  | P-value | Fold change |
| Lipid catabolism |  |  |  |  |  |
| BCG_3636 | Rv3571 | 3-ketosteroid-9-alpha-hydroxylase reductase subunit possibly involved in lipid and cholesterol catabolism | KshB | 0.01477 | 13.52217396 |
| BCG_3586 | Rv3522 | Lipid transfer protein or keto acyl-CoA thiolase, probably involved in lipid metabolism | Ltp4 | 0.01838 | 2.84084021 |
| BCG_1253 | Rv1193 | Fatty-acid-CoA ligase | FadD36 | 0.03509 | 1.21926237 |
| BCG_3162 | Rv3139 | Acyl-CoA dehydrogenase | FadE24 | 0.03903 | 0.656746417 |
| PDIM/PGL biosynthesis |  |  |  |  |  |
| BCG_2957 | Rv2935 | Phenolpthiocerol synthesis type-I polyketide synthase | PpsE | 0.00081 | 2.574516 |
| BCG_2961 | Rv2939 | Phthiocerol/phthiodiolone dimycocerosyl transferase | PapA5 | 0.01204 | 3.6804062 |
| BCG_2955 | Rv2933 | Phthiocerol synthesis polyketide synthase type I | PpsC | 0.0287 | 9.091669627 |
| Lipid anabolism |  |  |  |  |  |
| BCG_0877c | Rv0824c | Acyl-acyl carrier protein desaturase, possibly involved in mycolic acid synthesis | DesA1 | 0.00312 | 1.797705722 |
| BCG_2812c | Rv2794c | Phosphopantetheinyl transferase involved in lipid (such as mycolic acid) synthesis | PptT | 0.00363 | 0.308824302 |
| BCG_3863c | Rv3801c | Long-chain-fatty-acid--AMP ligase, involved in mycolic acid biosynthesis. | FadD32 | 0.02558 | 0.630710789 |
| BCG_2545c | Rv2524c | Fatty acid synthase | Fas | 0.04625 | 0.720299414 |
| DosR regulon |  |  |  |  |  |
| BCG_2653c | Rv2626c | Hypoxic response protein | Hrp1 | 0.00611 | 3.388341454 |
| BCG_2013 | Rv1996 | Universal stress protein | NA | 0.02264 | 2.39536029 |
| BCG_2051 | Rv2032 | NAD(P)H nitroreductase | Acg | 0.02359 | 3.289380911 |
| BCG_3155c | Rv3132c | Two component sensor histidine kinase | DevS/DosS | 0.03576 | 2.420244647 |
| Transcription/translation |  |  |  |  |  |
| BCG_2862c | Rv2842c | Ribosome maturation factor | RimP | 0.00426 | 0.248842399 |
| BCG_3648c | Rv3583c | RNA polymerase-binding transcription factor | CarD | 0.01658 | 0.703739813 |
| BCG_3521c | Rv3456c | 50S ribosomal protein L17 | RplQ | 0.02097 | 0.679231021 |
| BCG_2947c | Rv2925c | Ribonuclease III, involved in the processing of ribosomal RNA precursors and some mRNAs | Rnc | 0.02851 | 1.901367777 |
| BCG_0289c | Rv0251c | Heat shock protein able to bind to 30S ribosomal subunit and possibly involve translation under stress conditions | Hsp | 0.0293 | 0.183587219 |
| BCG_2578c | Rv2555c | Alanine--tRNA ligase | AlaS | 0.0307 | 2.641757666 |
| BCG_2925c | Rv2904c | 50S ribosomal protein L19 | RplS | 0.0325 | 0.248801917 |
| BCG_2930c | Rv2909c | 30S ribosomal protein S16 | RpsP | 0.03318 | 0.684450654 |
| BCG_0084 | Rv0053 | 30S ribosomal protein S6M binding with S18 to 16S ribosomal RNA | RpsF | 0.04228 | 0.656813947 |
| BCG_1358 | Rv1298 | 50S ribosomal protein L31 | RpmE | 0.04302 | 0.77699772 |
| BCG_3508c | Rv3442c | 30S ribosomal protein S9 | RpsI | 0.0473 | 0.651466304 |
| TCA |  |  |  |  |  |
| BCG_1300 | Rv1240 | Malate dehydrogenase | Mdh | 0.04198 | 0.642891999 |
| Others |  |  |  |  |  |
| BCG_1343 | Rv1284 | Beta-carbonic anhydrase | CanA | 0.00374 | 2.021655775 |
| BCG_1698 | Rv1659 | Probable argininosuccinate lyase involved in arginine biosynthesis (last step) | ArgH | 0.00406 | 0.485682093 |
| BCG_0381 | Rv0342 | Isoniazid inductible protein | IniA | 0.0197 | 0.642487573 |
| BCG_2597 | Rv2574 | It may have a deep hydrophobic ligand-binding pocket and participate in the periplasmic electron-transport chain | Hypothetical protein | 0.03222 | 0.73615781 |
| BCG_0056 | Rv0025 | It harbors conserved domain similar to component of the type VI protein secretion system | Hypothetical protein | 0.03494 | 0.489363989 |
| BCG_0382 | Rv0343 | Isoniazid inductible gene protein | IniC | 0.04183 | 0.633342781 |
| BCG_0140c | Rv0107c | Cation-transporter ATPase I, possibly catalyzes the transport of a cation (possibly magnesium) with the hydrolysis of ATP | CtpI | 0.04283 | 0.337580777 |
| BCG_3238 | Rv3211 | ATP-dependent RNA helicase | RhlE | 0.04392 | 1.912277818 |
| BCG_0240 | Rv0203 | Possible mycobacteria-type hemophore, which are secreted heme-binding proteins involved in heme acquisition. | Hypothetical protein | 0.04582 | 0.157337495 |
| BCG_0322 | Rv0282 | ESX-3 type VII secretion system protein | EccA3 | 0.04834 | 0.663212944 |
| BCG_1665c | Rv1627c | Nonspecific lipid-transfer protein, possibly involved in lipid metabolism | NA | 0.04911 | 2.819121315 |
| BCG_0159 | Rv0125 | Serine protease, possibly hydrolyzing peptides and/or proteins after serine residues | PepA | 0.04948 | 1.407853906 |
